# CBC Complex Regulates Hyphal Growth, Sclerotial Quantity, and Pathogenicity in the Necrotrophic Fungus *Botrytis cinerea*

**DOI:** 10.3390/jof11060429

**Published:** 2025-06-02

**Authors:** Yinshan Zhang, Xueting Chen, Guihua Li, Qingming Qin, Mingzhe Zhang, Jianchun Qin

**Affiliations:** College of Plant Science, Jilin University, Changchun 130062, China; zhangyinshan21@126.com (Y.Z.); 17688532553@163.com (X.C.); liguihua@jlu.edu.cn (G.L.); qmqin@jlu.edu.cn (Q.Q.)

**Keywords:** cap-binding protein complex, *BcCBP20*, *BcCBP80*, RRM, MIF4G, infection cushion, stress response, *B. cinerea*

## Abstract

The cap-binding protein complex (CBC), comprising Cbp20 and Cbp80, is crucial for gene expression, yet its role in the notorious crop pathogen *Botrytis cinerea* remains unclear. Immunoprecipitation coupled with LC-MS/MS demonstrated that BcCbp20 interacts with BcCbp80. Yeast two-hybrid, GST pull-down, and Split-luciferase complementation assays confirmed that the conserved RNA recognition motif (RRM, 54–127 aa) of BcCbp20 and the N-terminal MIF4G domain (1–370 aa, 1–577 aa) of BcCbp80 constitute the core interaction regions. Genetic transformation experiments revealed that *BcCBP80* exerts a more dominant role than *BcCBP20* in regulating hyphal morphology, growth rate, conidiophore development, and conidial yield. Furthermore, *BcCBP20* and *BcCBP80* differentially regulate sclerotium formation to maintain sclerotial quantity. Based on pathogenicity assays, *BcCBP80* associated with infection cushion development, with this phenotypic alteration possibly being among the factors correlated with altered pathogenicity. However, the increased sensitivity of Δ*Bccbp20* to various stress factors may be the primary reason for the diminished pathogenicity. Taken together, these results indicate that *BcCBP20* and *BcCBP80* play important roles in multiple aspects of *B. cinerea* growth, development, stress response, and pathogenicity.

## 1. Introduction

Gray mold, caused by *Botrytis cinerea*, poses a significant threat to a diverse array of host plants, including strawberries, raspberries, and grapes, resulting in substantial annual economic losses globally [1]. Given the astonishing reproductive capacity and high genetic diversity of *B. cinerea*, the escalating resistance of this fungus to traditional fungicides poses a significant global challenge [2,3]. However, the completion of the genome sequencing of *B. cinerea* and the understanding of its pathogenic mechanisms not only provide a solid theoretical foundation for the development of novel fungicides but also introduce new strategies for addressing this long-standing challenge that has plagued agricultural production [4]. Previous studies have indicated that conidia, the primary infectious propagules, germinate on plant surfaces and subsequently develop appressoria-like structures to facilitate penetration into the host [5,6]. Additionally, in this pathogen, highly melanized specialized hyphal networks, known as “infection cushions,” are formed, emanating from mycelia as another critical invasion strategy [5,7]. *B. cinerea* also secretes a diverse array of cell wall-degrading enzymes, extracellular proteins, and secondary metabolites, which collectively augment the efficiency of plant tissue penetration and colonization, ultimately enhancing virulence [8,9,10]. Upon successful colonization, the fungus exploits nutrients derived from necrotic host tissue to produce sclerotia, ensuring long-term survival under adverse environmental conditions, while also facilitating reinfection. The germination of sclerotia results in the development of mycelia and conidiophores, which subsequently produce a substantial number of conidia. These conidia are disseminated by natural elements, such as wind and rain, as well as agricultural implements, enabling their adhesion to plant surfaces and the initiation of a new infection cycle [1,5].

Cap-Binding Protein 20 and 80 (Cbp20 and Cbp80), also known as Ncbp2/Nip1 and Ncbp1/Ncbp, respectively, constitute the core components of the Cap-Binding protein Complex (CBC) in eukaryotes [11]. This complex plays a pivotal role in various aspects of RNA metabolism, including pre-mRNA splicing [12], microRNA maturation [13,14], mRNA stability [15,16], and mRNA export from the nucleus [17,18]. In contrast to prokaryotes, eukaryotic mRNAs possess a 5’ cap structure, which is specifically recognized and bound by CBC, facilitating efficient nuclear export and subsequent translation [11,19].

CBC is a highly conserved protein complex found across a broad spectrum of species. Numerous experiments are being conducted to investigate the role of Cbp20 and Cbp80 in diverse biological processes, ranging from yeast to human pathogens and plants.

As evidenced by previous studies, in *Saccharomyces cerevisiae*, *CBP80* and *CBP20* are not absolutely necessary for cell survival [20], but they are indeed crucial for supporting cell growth and proliferation [21]. Additional experiments have delved into the mechanisms underlying *CBP20*/*80*’s regulation of gene expression. Notably, in yeast, the *SUS1* gene (critical for histone H_2_B over-ubiquitination) relies on the precise splicing of its pre-mRNA to maintain proper chromatin modification. The loss of CBC disrupts *SUS1* splicing, leading to reduced Sus1 protein, the accumulation of over-ubiquitinated H_2_B, and altered chromatin structure and gene expression [22]. CBC also plays an important role in the pathogenic mechanisms of pathogens of global concern. Studies demonstrate the following: *SARS-CoV-2* synthesizes m7GTP via its viral enzyme Nsp14 to block CBC-mRNA cap interactions, thereby suppressing nuclear export and host gene expression [23]; *HIV-1* recruits the CBP80/NCBP3 complex and host RNA helicase A (RHA) to assemble an eIF4E/mTOR-independent translation initiation complex, enabling persistent viral protein synthesis under host translational inhibition [24]; viral mRNAs hijack host mRNA processing pathways (splicing, nuclear export, and translation) to facilitate viral proliferation [25].

In plants, research has primarily focused on the interplay between *CBP20*/*80* and stress responses. In the model plant *Arabidopsis thaliana*, the disruption of CBC genes led to delayed development and the downregulation of transcripts essential for abscisic acid (ABA) signaling pathways, thereby enhancing sensitivity to the hormone ABA [26]. Kong et al. demonstrated that *CBP20*/*80* functions as a mediator in the splicing of *P5CS1* and *IDD14* genes, intricately regulating their expression levels, which subsequently caused significant alterations in plant proline and starch content, ultimately enhancing resilience and tolerance to salt stress [27]. Li further showed that SR45a-1a, a conserved serine/arginine-rich (SR)-like protein, directly interacts with Cbp20 to mediate responses to salt stress. Additionally, the truncated isoform SR45a-1b facilitates SR45a-1a binding to Cbp20 [28]. Zhang revealed that ethylene governs *CBP20* phosphorylation, a critical process for the upregulation of miR319b and downregulation of its target gene, *MYB33* (a MYB transcription factor involved in plant growth, development, metabolism, and environmental responses). This cascade ultimately culminates in ethylene-induced root growth inhibition [29].

Although *CBP20* and *CBP80* have been extensively characterized in human pathogens and model organisms, their functional significance in plant pathogenic fungi remains poorly understood, particularly regarding the interaction patterns of the CBC in *B. cinerea*, the roles of its constituent members during vegetative growth processes, and the molecular mechanisms modulating pathogenicity. To address these questions, this study integrates a bioinformatics analysis, targeted gene deletion, and phenotypic virulence assays to elucidate the cooperative mechanisms and functional specialization between *BcCBP20* and *BcCBP80* in the pathogenesis of *B. cinerea*, a globally devastating phytopathogen. The findings not only advance the mechanistic understanding of plant–*B. cinerea* interactions but also establish a theoretical framework for novel fungicide development.

## 2. Materials and Methods

### 2.1. Acquisition of Sequences, Alignment, Prediction of Functional Domains, and Construction of Phylogenetic Trees

The complete genomic sequences encompassing *BcCBP20* (GenBank accession XP_001557145, accessed on 6 November 2023) and *BcCBP80* (GenBank accession XP_024553977, accessed on 6 November 2023), along with their respective 5′-/3′-flanking regions and promoter sequences, were systematically retrieved through NCBI GenBank (https://www.ncbi.nlm.nih.gov/ accessed on 6 November 2023). The sequences utilized in this study have been provided in Supplementary Table S1.

The amino acid sequences of homologous genes were subjected to BLASTP with the NCBI database (https://blast.ncbi.nlm.nih.gov/Blast.cgi?PROGRAM=blastp&PAGE_TYPE=BlastSearch&LINK_LOC=blasthome accessed on 6 November 2023). The protein domains were predicted using the SMART website (http://smart.embl.de/ accessed on 6 November 2023) and visualized with DOG 2.0 software (https://gps.biocuckoo.cn/download.php accessed on 6 November 2023). Amino acid sequences of Cbp20/Cbp80 from different organisms were aligned with DNAMAN 5.0 software (https://www.lynnon.com/dnaman.html accessed on 6 November 2023) Homologous sequences of *CBP20*/*CBP80* from target species were retrieved from the NCBI database. The initial alignment utilized full-length amino acid sequences of the target proteins, followed by manual trimming to remove low-quality alignment regions. A phylogenetic tree was constructed using MEGA 6 software with the neighbor-joining (NJ) method and maximum-likelihood (ML) distance composite model, supported by 1000 bootstrap replicates [30].

### 2.2. Fungal Transformation, Mutant Generation, and Characterization

*BcCBP20* and *BcCBP80* deletion mutants, designated as Δ*Bccbp20* and Δ*Bccbp80*, respectively, along with their genetically complemented strains (Δ*Bccbp20*-C and Δ*Bccbp80*-C) were generated as described in [31,32]. Briefly, the genomic DNA extracted from mycelia harvested from potato dextrose agar (PDA) medium (Coolaber, PM0520, Beijing, China) using a previously described method served as the template for these PCR reactions [33,34]. The upstream and downstream flanking regions of each gene were amplified using the specific primer pairs 20-Up-F/20-Up-R and 20-Down-F/20-Down-R for *BcCBP20*, and 80-Up-F/80-Up-R and 80-Down-F/80-Down-R for *BcCBP80*. The recombinant plasmid was constructed by assembling PCR products into the pXEH-TK2 vector through homologous recombination using the ClonExpress II One-Step Cloning Kit (Vazyme, C112-01, Nanjing, China) following the manufacturer’s protocol. The pXEH-TK2 vector was constructed by inserting the codon-optimized thymidine kinase (*HSVtk(Bc)*, GenBank accession PQ362314) into the pXEH backbone [35,36,37]. The knockout constructs were introduced into *Agrobacterium tumefaciens* AGL-1 via electroporation, and the transformed AGL-1 cells were then used to transfer the constructs into the B05.10 strain through *Agrobacterium tumefaciens*-mediated transformation (ATMT) [38]. Following transformation, the transformants were selected on media plates containing hygromycin and 5-fluorouracil 2′-deoxyriboside. For genetic complementation, the primer pairs C-20-F/C-20-R and C-80-F/C-80-R were used to amplify sequences encompassing the promoter and full length of the *BcCBP20* and *BcCBP80* genes, respectively. These fragments were cloned into the pXEG vector [39]. The successful complementation vectors were subsequently introduced into the Δ*Bccbp20* and Δ*Bccbp80* strains using the same transformation protocol. The complemented strains were selected on PDA plates containing G418.

A diagnostic PCR was performed to verify the integration events of the selected transformants. The deletion mutants and the complemented strains were further validated via a Reverse-Transcription–Polymerase Chain Reaction (RT-PCR) analysis. The primers used in those experiments are listed in Supporting Information Table S1.

### 2.3. RT-PCR

The total RNA was extracted from mycelium samples of *B. cinerea* using the RNAiso Plus reagent (TaKaRa, 9108, Beijing, China). An amount of 1 μg of RNA from each strain was used for first-strand cDNA synthesis, employing the PrimeScript^®^ RT Reagent Kit (TaKaRa, RR036A, Beijing, China). SYBR^®^ Green I (TaKaRa, CN830A, Beijing, China) dyes were used for the RT-PCR analysis. The pathogen actin gene, *BcACT1*, was used as an endogenous reference.

### 2.4. Generation of BcCbp20::GFP:B05.10 Strain, Western Blot Validation, and Mass Spectrometric Analysis

The *oliC* promoter (amplified from the pCB1532 vector; GenBank accession PP209073 3246–4286 bp), *BcCBP20* gene (amplified from B05.10 cDNA), and C-terminal GFP tag (amplified from pCB1532 vector, 4307–5077bp) were ligated into the XhoI/KpnI-linearized pXEH vector using the ClonExpress II One-Step Cloning Kit (Vazyme, C112, Nanjing, China). The primers used are listed in Table S1. After Sanger sequencing verification by Sangon Biotech (Shanghai, China), the construct was transformed into B05.10 via ATMT. Hygromycin-resistant transformants were selected. Mycelia were cultured on complete medium (CM) plates [40], and total proteins were extracted using a lysis buffer (Beyotime, P0013, Shanghai, China; containing 20 mM Tris-HCl [pH 7.5], 150 mM NaCl, 1% Triton X-100) and supplemented with protease inhibitor cocktail (Sangon Biotech, C600382, Shanghai, China) and phenylmethylsulfonyl fluoride (PMSF, Sangon Biotech, A460281, Shanghai, China). Total proteins were incubated with GFP-Trap^®^ magnetic beads (Proteintech, 17333363, Rosemont, IL, USA) at 4 °C overnight, followed by three washes with the wash buffer (20 mM Tris-HCl, 500 mM NaCl, pH 7.5). A Western blot analysis was performed as previously described [41,42]. Total proteins (10 μL) and eluted proteins (10 μL) were resolved on 10% SDS-PAGE gels, electrophoretically transferred to PVDF membranes (Sigma, IPFL00005, St. Louis, MO, USA), and immunoblotted with an anti-GFP antibody (Proteintech, HRP-66002, Rosemont, IL, USA). Chemiluminescent signals were captured using an Amersham Imager 600 system. The remaining proteins released from the beads were digested and analyzed via liquid chromatography–tandem mass spectrometry (LC-MS/MS) at KangChen Bio-tech (Shanghai, China). Raw data were processed with MaxQuant 2.0.1.0 against the *B. cinerea* UniProt database (UP000001798), with the following filtering criteria: expression fold difference: ratio A/B ≥ 2.0, unique peptides ≥ 2. Further details of the experimental principles and operational procedures can be found at https://www.aksomics.com/services/proteomics/lfq.html (accessed on 10 April 2024).

### 2.5. Yeast Two-Hybrid Assay

The Y2H assays were performed as previously described [42,43,44]. Briefly, the in-frame expression cDNA of candidate genes was amplified and subcloned into pGBKT7 (Clontech, 630443, San Jose, CA, USA) or pGADT7 (Clontech, 630442, San Jose, CA, USA) via homologous recombination using the Vazyme C112-01 kit (primers listed in Table S1). All bait–prey combinations (including mock controls) were co-transformed into AH109 yeast cells (*MATa*, Weidi, YC1010, Shanghai, China) following the Matchmaker Gold Yeast Two-Hybrid System manufacturer instructions (Clontech, 630489, San Jose, CA, USA). Transformants were then plated onto synthetic-defined (SD) double-dropout medium (DDO: -Leu/-Trp; Weidi, YM3200L, Shanghai, China). The yeast cell from the DDO plate was inoculated into the DDO liquid medium and cultured until reaching an OD_600_ of 0.75. Subsequently, 5 μL of serially diluted cell suspensions (10^−1^ to 10^−3^) was spotted onto the SD quadruple dropout medium (QDO: -Leu/-Trp/-His/-Ade; Weidi, YM3402L, Shanghai, China) and incubated at 30 °C for 2–3 days to assess the protein–protein interactions.

### 2.6. GST Pull-Down Analysis

The GST pull-down assays were conducted following the previously established protocol [45,46]. Briefly, the in-frame expression cDNA of candidate genes was amplified and subcloned into pET21b (Sigma-Aldrich, 69741-3, St. Louis, MO, USA) or pGEX6p-1 (Sangon Biotech, AA339094, Shanghai, China) via homologous recombination (primers listed in Table S1). The fusion construct was expressed in *E. coli* BL21 (DE3) (Zoman, ZK201, Beijing, China) [47], and proteins were extracted through ultrasonication. GST fusion proteins (equal concentrations) were mixed with His fusion proteins (specified amounts) and supplemented with 1×PBS buffer to a final volume of 2 mL. An amount of 20 μL of pre-rinsed Glutathione Sepharose beads (GE Healthcare) (Coolaber, CS20421, Beijing, China) was added, and the mixture was incubated at room temperature for 1 h with gentle agitation. Beads were washed 5–7 times with the wash buffer, followed by resuspension in 1×SDS sample loading buffer and boiling for 5 min prior to SDS-PAGE. A Western blot analysis was performed using monoclonal anti-GST (Proteintech, HRP-66001, Rosemont, IL, USA) and anti-His (Proteintech, HRP-66005, Rosemont, IL, USA) antibodies.

### 2.7. Split-Luciferase Complementation

The SLC assays were performed as previously described [43,44]. Briefly, *Agrobacterium* strain EHA105 (Zoman, ZK294, Beijing, China) cultures containing the appropriate constructs were adjusted to OD_600_ = 0.8–1.0 using a buffer composed of 10 mM MgCl_2_, 10 mM MES (pH 5.6), and 200 μM acetosyringone (AS). The cultures were transiently infiltrated into leaves of *Nicotiana benthamiana* (Nb). Forty-eight hours post-infiltration (hpi), leaves were sprayed with 1 mM D-luciferin (Sangon Biotech, A422342, Shanghai, China), and the luciferase activity was visualized using a cooled CCD imaging system (Tanon 5200). For relative activity quantification, two 28 mm^2^ leaf disks were immersed in 200 μL of 1 mM luciferin, and luminescence was measured using a GloMax 96-Microplate Luminometer Promega (E&K Scientific EK-25075, CA, USA).

### 2.8. Mycelial Growth, Sporulation, Germination, Conidiophores, and Sclerotium Formation

A 5 mm diameter mycelial plug was taken from the edge of each test strain and inoculated onto PDA and CM plates, respectively. The plates were incubated in the dark at 20 °C. Images were captured, and fungal diameters were recorded on the third day. Additionally, a 5 mm diameter mycelial plug from each tested strain was placed on a glass slide, and the mycelial morphology was observed under a Nikon microscope after 24 h. Conidiophore development was assessed three days later.

Mycelial plugs from each test strain were inoculated onto CM plates. After 14 days of incubation, conidia were collected and diluted to a volume of 1 mL. The number of conidia produced by each strain was recorded, and conidia were observed and photographed for documentation. Fresh spore suspensions were adjusted to 5 × 10^5^ spores/mL, mixed with an equal volume of the CM containing 200 mg/L streptomycin, and 10 µL of the mixture was transferred to a glass slide. The slides were incubated at 20 °C in the dark for 2, 4, or 6 h, after which the germination rates were analyzed microscopically. Images were captured, germ tube lengths were measured, and the percentage of germinated conidia was calculated.

After 30 days of incubation on CM plates, sclerotia formation was observed and photographed. The size and quantity of sclerotia were recorded. Subsequently, sclerotia were inoculated onto CM plates to assess the germination rates and measure the mycelial growth length.

### 2.9. The Formation of Appressoria and Development of Infection Cushions

The experimental procedures were consistent with the aforementioned spore germination assay. At 8 h post-inoculation, the appressoria formation in the strains B05.10, Δ*Bccbp20*, and Δ*Bccbp20*-C was analyzed microscopically. Images were captured, and the percentage of appressoria formation was quantified for each strain. The infection cushion formation was examined at 24, 36, and 48 hpi, with microscopic images documented. A 5 mm diameter mycelial plug from each strain (B05.10, Δ*Bccbp80*, and Δ*Bccbp80*-C) was transferred to a glass slide. Infection cushions were observed under a microscope at 56 hpi.

### 2.10. Plant Infection Assays

The fresh spore suspension of B05.10, Δ*Bccbp20-4*, Δ*Bccbp20-6*, and Δ*Bccbp20*-C were adjusted to 1×10^6^ spores/mL. Each spore suspension was mixed with an equal volume of liquid CM, and 20 μL aliquots of the mixture were inoculated onto green bean leaves (*Phaseolus vulgaris*) and tobacco leaves (*Nicotiana benthamiana*). Alternatively, a 5 mm diameter fungal plug from B05.10, Δ*Bccbp20*, Δ*Bccbp20*-C, Δ*Bccbp80*, and Δ*Bccbp80*-C were removed from the edge of the mycelium and inoculated onto freshly detached bean leaves, tobacco leaves, and the apricot cultivar ‘Jintaiyang’ (Prunus armeniaca var. ansu). Images were captured at the designated time points, and the lesion areas were quantified.

### 2.11. Stress Adaptation Assays

A 5 mm diameter mycelial plug from each test strain was inoculated onto CM plates supplemented with various stress agents, including osmotic stress agents (0.6 M NaCl and 0.6 M KCl) (Sangon Biotech, A610476, A610440, Shanghai, China), an oxidative stress agent (7.5 mM H_2_O_2_) (Nanjing Reagent, 7722-84-1, Nanjing, China), and cell wall perturbation (0.005% SDS and 500 μg/mL Congo Red) (Sangon Biotech, A600485, A600324, Shanghai, China). Plates were incubated in the dark at 20 °C. Mycelial growth was measured and photographed after 2.5 days of incubation. All experiments were conducted in triplicate (three technical replicates per treatment). The adaptability to stress agents was assessed by comparing colony diameters after incubation, and temperature sensitivity was evaluated by culturing test strains in the dark at both 20 °C and 28 °C. Mycelial growth was measured and photographed after 3 days of incubation. All experiments were conducted in triplicate (three technical replicates per treatment) and repeated three times independently (three biological replicates).

### 2.12. Statistical Analysis

All the quantitative data in this study were derived from the results of at least three independent experiments. The quantity of spores, AP, and ICs was determined through manual counting, while their lengths, areas, and lesion sizes were measured using ImageJ software 1.46r (https://imagej.net/ij/ accessed on 12 November 2023). To compare differences in quantitative data, such as mycelial growth, lesion size, and conidial production, between different strains, the observed value of the B05.10 was set to 100%, and other strains were normalized accordingly. For the normalization of time series data, the final time point of the WT strain was used as the 100% reference point. Statistical significance between groups (control, knockout, complemented strains) was determined using Student’s *t*-test, with a threshold of *p* < 0.05.

## 3. Results

### 3.1. Identification BcCbp20/BcCbp80 in B. cinerea

The role of cap-binding proteins 20/80 (Cbp20/Cbp80) in the crop killer *B. cinerea* has captured our attention. A bioinformatics analysis revealed that the *BcCBP20* gene (676 bp) contains three exons and two introns, encoding a 181-amino acid (aa) protein, while *BcCBP80* (3286 bp) consists of four exons and three introns, encoding an 876-aa protein. A domain prediction analysis revealed that *CBP20* orthologs contain an RNA recognition motif (RRM) domain, while *CBP80* orthologs possess MIF4G, MIF4G_like, and MIF4G_like_2 domains, where MIF4G corresponds to the middle domain of eukaryotic initiation factor 4G (eIF4G). *P. oryzae* lacks the MIF4G domain (Figures S1A and S2A).

A comparative analysis revealed that the full-length BcCbp20 amino acid sequence shares sequence identity with Cbp20 orthologs from *Sclerotinia sclerotiorum* (XP_001585390.1, 98.90%), *Aspergillus nidulans* (XP_663746.2, 77.97%), *Fusarium graminearum* (XP_011324033.1, 77.22%), *Neurospora crassa* (XP_956595.2, 77.01%), *Pyricularia oryzae* (XP_003717292.1, 75.58%), *Trichoderma reesei* (XP_006967414.1, 74.42%), *Homo sapiens* (NP_031388.2, 66.41%), *Drosophila melanogaster* (NP_524396.1, 65.00%), *Caenorhabditis elegans* (NP_001370288.1, 63.87%), *Arabidopsis thaliana* (AAO44079.1, 58.15%), and *Saccharomyces cerevisiae* (NP_015147.1, 54.14%) (Figure S1B). The full length of BcCbp80 shares sequence identities of 85.03%, 60.42%, 59.03%, 58.21%, 56.45%, 53.24%, 24.34%, 23.51%, 23.21%, 22.69%, and 21.75% with Cbp80 orthologs from the following corresponding species: *S. sclerotiorum* (APA15411.1), *N. crassa* (XP_961147.1), *T. reesei* (XP_006961102.1), *A. nidulans* (KAL4766915.1), *F. graminearum* (XP_011327077.1), *P. oryzae* (XP_003719884.1), *S. cerevisiae* (NP_013844.2), *D. melanogaster* (NP_726938.1), *H. sapiens* (BAD92470.1), *A. thaliana* (NP_565356.1), and *C. elegans* (NP_491850.2) (Figure S2B).

Phylogenetic tree analysis revealed that the Cbp20/Cbp80 proteins of *B. cinerea* and *S. sclerotiorum*, which cluster within a single clade, exhibit the closest evolutionary relationship, followed by those of other filamentous fungi (Figures S1A and S2A).

### 3.2. Identification of Interacting Proteins and Subcellular Localization of the CBC Component BcCbp20 in B. cinerea

**Figure 1:**
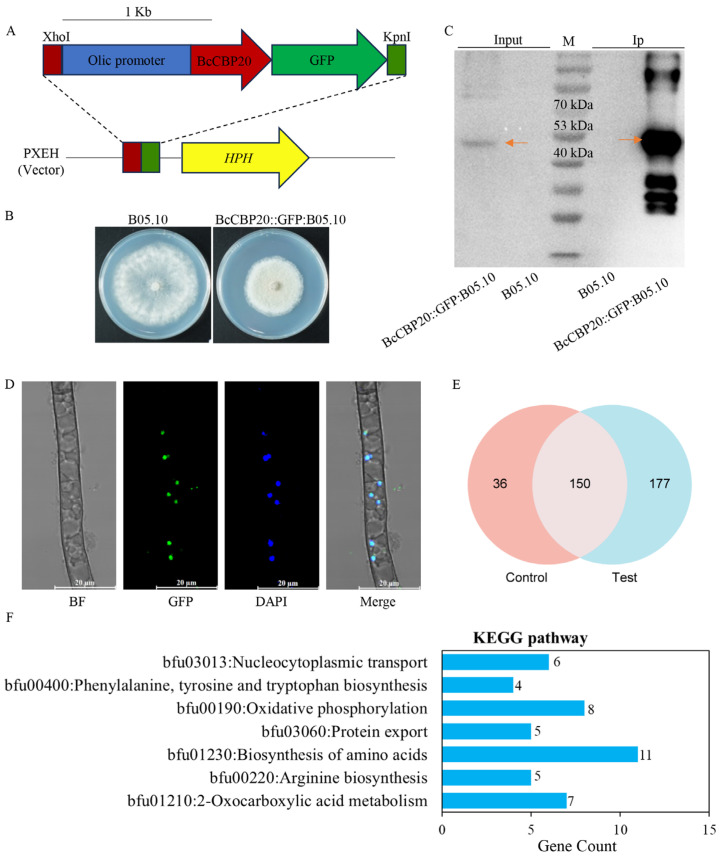
BcCbp20 interactome and nuclear localization in *B. cinerea.* (**A**) Schematic diagram of the BcCBP20::GFP fusion construct driven by the *Olic* promoter. (**B**) Mycelial growth of transformant BcCBP20::GFP:B05.10 and WT on PDA plates after 3 days of incubation in the dark. (**C**) Validation of BcCBP20::GFP protein expression. Western blot analysis of total proteins (Input) and affinity-purified proteins (IP) from WT and BcCBP20::GFP:B05.10 transgenic strain Anti-GFP antibody detected 48 kDa bands (as indicated by red arrows) in both BcCBP20::GFP-Input and BcCBP20::GFP-IP samples, with no signal in WT controls. M: Protein molecular weight marker. (**D**) Nuclear localization of BcCBP20::GFP fusion protein. BF: bright field, BcCBP20-GFP signal (green), DAPI nuclear staining (blue), Merged image (BF+GFP+DAPI). (**E**) Venn diagram of LC-MS/MS-identified proteins in GFP pull-down assays (test: BcCBP20::GFP:B05.10; control: WT). (**F**) KEGG pathway enrichment analysis of 177 BcCBP20-associated specific proteins, revealing key roles in biosynthesis of amino acids, oxidative phosphorylation, 2-oxocarboxylic acid metabolism, nucleocytoplasmic transport, arginine biosynthesis, protein export, and phenylalanine, tyrosine, and tryptophan biosynthesis.

In eukaryotic cells, CBC is a well-conserved heterodimer from yeast to humans, composed of *CBP20* and regulatory subunit *CBP80*, which binds nascent RNA polymerase II transcripts and coordinates multiple RNA metabolic processes. To investigate CBC component interactions in *B. cinerea*, we engineered a *GFP*-tagged *BcCBP20* construct under the control of the *Olic* promoter in the WT strain (Figure 1A,B). A Western blot analysis under denaturing conditions (10% SDS-PAGE) confirmed BcCBP20::GFP expression in the B05.10 transformant and validated the affinity-eluted proteins using GFP-tag magnetic beads (Figure 1C). A subcellular localization analysis in the BcCbp20::GFP:B05.10 strain revealed nuclear enrichment, as demonstrated by confocal microscopy showing the co-localization of GFP signals with DAPI-stained nuclei (Figure 1D). For pull-down assays, WT (control) and BcCbp20::GFP:B05.10 (test) strains were analyzed. LC-MS/MS identification detected 327 proteins in the test group and 186 in controls. After removing 151 nonspecific proteins bound to GFP beads, 177 strain-specific interactors were identified, including *BcCBP20* and *BcCBP80* (Figure 1E and Table S2). A KEGG pathway analysis revealed enrichment in the biosynthesis of amino acids, oxidative phosphorylation, 2-oxocarboxylic acid metabolism, nucleocytoplasmic transport, arginine biosynthesis, protein export, and phenylalanine, tyrosine, and tryptophan biosynthesis (Figure 1F).

### 3.3. BcCbp20-BcCbp80 Interaction in B. cinere Relies on the N-Terminal MIF4G Domain of BcCbp80

**Figure 2:**
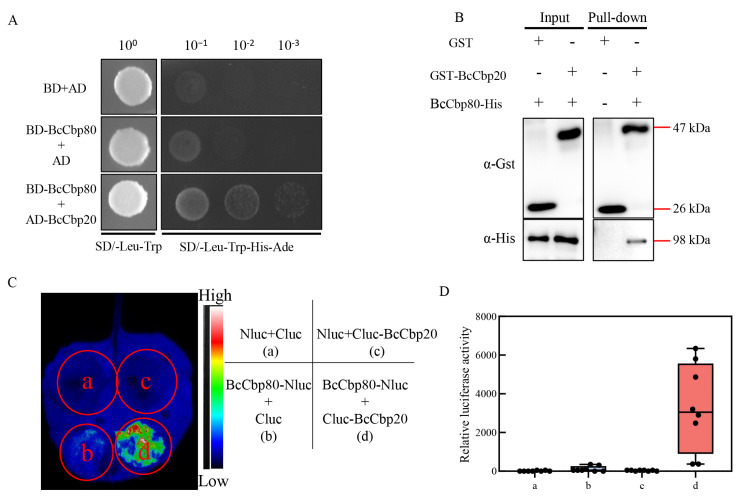
BcCbp20 physically interacts with BcCbp80. (**A**) Y2H confirmation of BcCbp20-BcCbp80 interaction. BcCbp80 was subcloned into pGBKT7 (BD) as bait, while BcCbp20 was sub-cloned into pGADT7 (AD) as prey. Co-transformed yeast strain AH109 was cultured on SD-Leu-Trp double-dropout medium. Interactions were tested on SD-Leu-Trp-His-Ade quadruple-dropout medium using serial dilutions (10^−1^ to 10^−3^). (**B**) GST pull-down assay confirming BcCbp20-BcCbp80 binding. cDNAs of BcCbp80 and BcCbp20 were subcloned into pET21b and pGEX6p-1, respectively. Fusion proteins expressed in *E. coli* BL21(DE3) were sonicated and used as input. BcCbp80-His and BcCbp20-GST were incubated with glutathione sepharose beads for immunoprecipitation (IP). Input and IP samples were analyzed by immunoblotting with anti-GST and anti-His antibodies, detecting GST (26 kDa), GST-BcCbp20 (47 kDa), and BcCbp80-His (98 kDa) (as indicated by red lines). +: signal detected by WB; −: no signal (**C**,**D**) Split-luciferase complementation (SLC) assays confirming the in vivo interaction. BcCbp20 and BcCbp80 cDNAs were subcloned into pNLUC and pCLUC, respectively. *Agrobacterium tumefaciens* EHA105 carrying these constructs were co-infiltrated into *Nicotiana benthamiana* leaves. Luminescence images (**C**) and relative luciferase activity (**D**) were quantified at 48 h post-agroinfiltration. a: Nluc+Cluc, b: BcCbp80-Nluc+Cluc, c: Nluc+Cluc-BcCbp20, d: BcCbp80-Nluc+ Cluc-BcCbp20.

Previous studies in *F. graminearum* identified a direct physical interaction between FgCbp80 and FgCbp20 through yeast two-hybrid (Y2H) screening [48]. Building on LC-MS/MS results, we employed a multi-method strategy to characterize the cap-binding complex formation in *B. cinerea*. A Y2H analysis demonstrated specific BcCbp20-BcCbp80 interaction (Figure 2A). GST pull-down assays biochemically confirmed direct binding between GST-fused BcCbp20 and His-fused BcCbp80 (Figure 2B). Furthermore, Split-luciferase complementation (SLC) assays validated interaction between the two *B. cinerea* proteins. (Figure 2C,D). To delineate the structural determinants of this interaction, we systematically generated five truncation mutants of BcCbp80 spanning its annotated functional domains (Figure S3D). Our findings revealed that two N-terminal fragments containing the first MIF4G functional domain-BcCbp80 ^1-370^ and BcCbp80 ^1-577^ exhibit direct and robust interactions with not only the full-length BcCbp20 but also its functional domains BcCbp20 ^54-127^. Conversely, C-terminal fragments (BcCbp80 ^271-577^, BcCbp80 ^560-876^, and BcCbp80 ^271-876^) showed no detectable interaction with BcCbp20 ^54-127^ (Figure S3A, B, and C). In summary, our findings demonstrate a conserved BcCbp20-BcCbp80 interaction in *B. cinerea*, mechanistically dependent on BcCbp80’s N-terminus binding to BcCbp20 functional domains.

### 3.4. The BcCBP20 and BcCBP80 Genes Are Involved in the Vegetative Growth of B. cinerea

The elevated sequence identity and conserved interaction network suggest that the CBP protein in *B. cinerea* likely exerts a regulatory role analogous to its homolog in *F. graminearum*. To elucidate the biological functions of this RNA-binding protein complex, we generated *BcCBP20* and *BcCBP80* knockout mutants (Δ*Bccbp20*, Δ*Bccbp80*) via homologous recombination (Figure S4A,C). Genetic complementation strains Δ*Bccbp20*-C and Δ*Bccbp80*-C were successfully reconstructed using native promoters (Figure S4B,D). According to the primer schematic diagram (Figure S4E,G), the deletion mutants and complemented strains were confirmed via PCR and RT-PCR analyses (Figure S4F,H). These results collectively demonstrate the successful integration of the genetic constructs.

**Figure 3:**
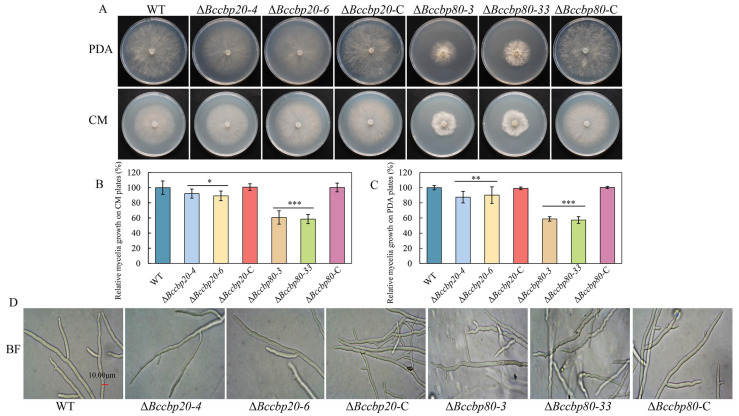
Growth, morphology, and statistical analysis of indicated strains on PDA and CM plates. (**A**) Fungal morphology of WT, Δ*Bccbp20-4*, Δ*Bccbp20-6*, Δ*Bccbp80-3*, Δ*Bccbp80-33*, Δ*Bccbp20*-C, and Δ*Bccbp80*-C strains was observed on PDA and CM plates after 72 h. (**B**,**C**) Quantification of relative growth of indicated strains on PDA or CM plates. *, **, ***: *p* < 0.05, 0.01, 0.001. Bar = 10 µm. (**D**) Hyphae of Δ*BcCbp80* mutants displayed abnormal curvature compared to WT and Δ*BcCbp80-C*.

Mycelial plugs from wild-type (WT), Δ*Bccbp20*, Δ*Bccbp80*, Δ*Bccbp20*-C, and Δ*Bccbp80*-C strains were inoculated on CM and PDA media. After 72 h incubation, radial growth measurements revealed severe growth impairment in Δ*Bccbp80* mutants, exhibiting a 60% reduction in diameter compared to WT and Δ*Bccbp80*-C. In contrast, Δ*Bccbp20* mutants maintained near-WT growth rates (90% of WT and Δ*Bccbp20*-C) (Figure 3A–C). A further microscopic examination revealed that Δ*Bccbp80* mutants exhibited curved hyphal morphology, in contrast to the straight hyphae observed in WT and Δ*Bccbp80*-C strains. Concurrently, Δ*Bccbp20* mutants showed no significant differences in hyphal morphology compared to WT and Δ*Bccbp20*-C strains (Figure 3D). These results demonstrate that *BcCBP80* plays a more pivotal role than *BcCBP20* in regulating hyphal morphogenesis and modulating mycelial nutritional growth.

### 3.5. BcCBP80 Is Essential for Conidiophore Development and Conidiation in B. cinerea

**Figure 4:**
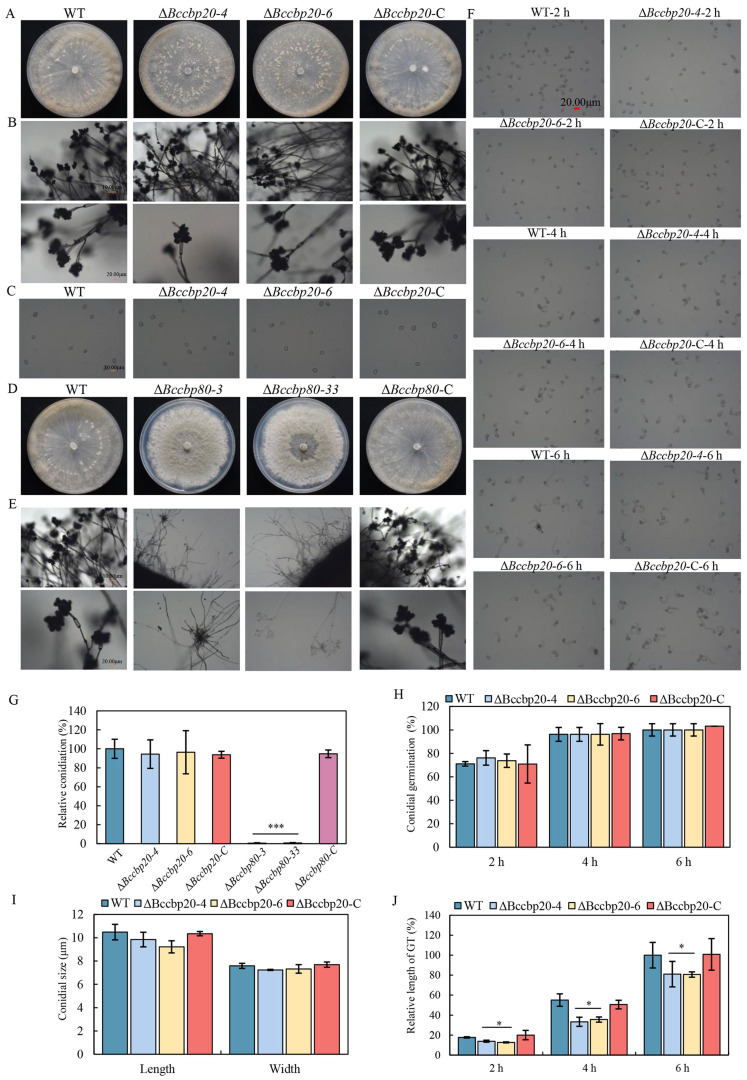
Conidiation, conidiophore morphology, conidial morphogenesis, and germination. (**A**) Conidiation of WT, Δ*Bccbp20-4*, Δ*Bccbp20-6*, and Δ*Bccbp20*-C incubated on CM plates for 14 days. (**B**) Conidiophore morphology of indicated strains. Scale bars = 10 µm (upper), 20 µm (lower). (**C**) Conidia morphology of the indicated strains. Scale bar = 10 µm. (**D**) Conidiation of WT, Δ*Bccbp80-3*, Δ*Bccbp80-33*, and Δ*Bccbp80*-C incubated on CM plates for 14 days. (**E**) Conidiophore of the indicated strains. Scale bars = 10 µm (upper), 20 µm (lower). (**F**) Conidial germination at indicated hpi. Scale bar = 20 µm. (**G**) Quantification of relative conidiation of the indicated strains on CM plates. *** indicates significance at *p* < 0.001. (**H**) Quantification of relative germination at the indicated hpi. (**I**) Conidial size of indicated strains. (**J**) Relative germ tube (GT) length at indicated hpi. *: *p* < 0.05.

The abundant conidia produced by *B. cinerea* during asexual reproduction are essential for disease spread. To elucidate the potential roles of *BcCBP20* and *BcCBP80* in this process, all strains were cultured on CM plates at 20 °C for 14 days (Figure 4A,D). A quantitative analysis revealed no significant difference in conidial production between Δ*Bccbp20*, WT, and Δ*Bccbp20*-C strains (Figure 4C,G). In contrast, Δ*Bccbp80* strains exhibited severe sporulation defects (Figure 4G).

Conidial morphology analysis revealed no significant variations in spore dimensions or morphology between the Δ*Bccbp20* mutants and control strains (Figure 4C,I). Similarly, conidiophore development in Δ*Bccbp20* mutants was comparable to WT and Δ*Bccbp20*-C strains (Figure 4B). The Δ*Bccbp80* mutant exhibits complete impairment in conidiophore morphogenesis (Figure 4E), with structural aberrations precluding conidiation initiation.

In addition, we observed spore germination at various time points. Our findings demonstrated no statistically significant difference in conidial germination rates among the tested strains (Figure 4H). However, at corresponding time points, the lengths of the germ tubes of Δ*Bccbp20-4* and Δ*Bccbp20-6* mutants were significantly shorter than those of the control strains, accounting for approximately 80–90% of the length observed in both the WT and Δ*Bccbp20*-C strains (Figure 4F,J).

In summary, *BcCBP80* exerts a greater influence on conidiophore and conidia production than *BcCBP20*.

### 3.6. BcCBP20 and BcCBP80 Play Distinct Roles in Sclerotium Formation

**Figure 5:**
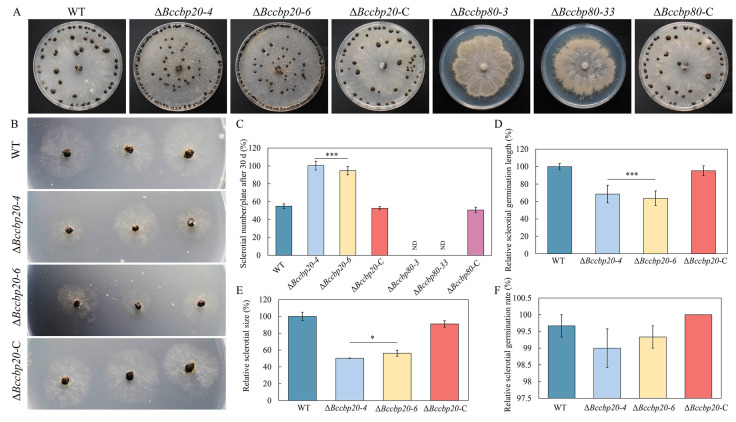
Sclerotium production and morphogenesis in Δ*Bccbp20*, Δ*Bccbp80* mutants. (**A**) Sclerotial formation of the indicated strains incubated on CM plates for 30 days. (**B**) Sclerotial germination of the indicated strains at 2 dpi. (**C**) Quantification of the relative sclerotial number per plate for the indicated strains. ***: *p* < 0.001. (**D**) Quantification of the relative sclerotial germination length of the indicated strains. ***: *p* < 0.001. (**E**) Quantification of the relative sclerotial size for the indicated strains. *: *p* < 0.05. (**F**) Quantification of the relative sclerotial germination rate of the indicated strains.

As parental tissues, sclerotia play important roles in both reinfection and stress survival. To investigate the role of CBC in sclerotial formation, mycelial plugs from each test strain were inoculated onto CM plates. After 30 days of incubation, the sclerotia were collected and analyzed (Figure 5A). Intriguingly, the Δ*Bccbp20* strains demonstrated a marked enhancement in sclerotial production compared to both the WT and the Δ*Bccbp20*-C strains (Figure 5C). Conversely, the Δ*Bccbp80* strains were completely incapable of producing sclerotia (Figure 5C). Additionally, the sclerotia produced by the Δ*Bccbp20* strains were only half the size of those of the WT and Δ*Bccbp20*-C strains (Figure 5E).

When we inoculated these sclerotia onto the CM (Figure 5B), we discovered that the smaller sclerotia from the Δ*Bccbp20* strains were capable of germinating normally (Figure 5F). A further analysis revealed that the mycelial lengths emanating from the sclerotia of Δ*Bccbp20* strains were approximately 70% of those of the WT and Δ*Bccbp20*-C strains (Figure 5D). Collectively, these results demonstrate that *BcCBP20* and *BcCBP80* play distinct roles in the process of sclerotium formation, ensuring the stability of sclerotium numbers.

### 3.7. BcCbp20 and BcCbp80 Are Required for Full Virulence in B. cinerea

**Figure 6:**
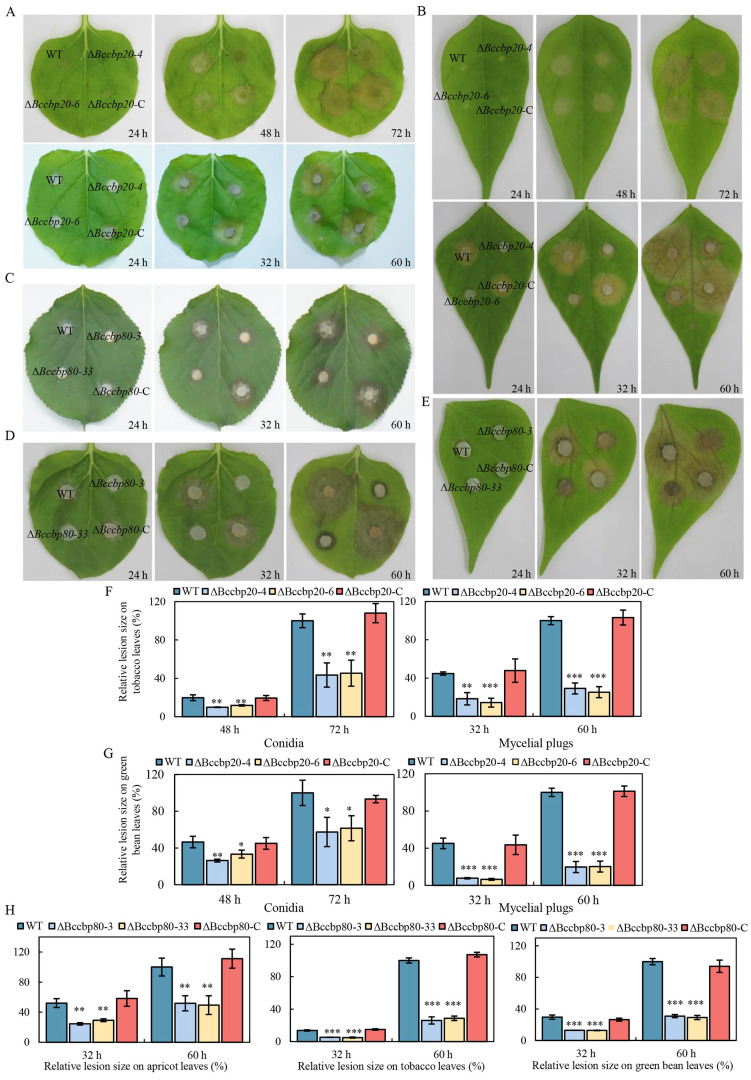
Pathogenicity test assay of indicated strains. (**A**) Diseased tobacco leaves caused by the indicated strains during infection time course. Upper panel: spore inoculation; lower panel: mycelial plug inoculation. (**B**) Diseased green bean leaves caused by the indicated strains during a time course of infection. Upper image: spore inoculation; lower panel: mycelial plug inoculation. (**C**–**E**) Diseased apricot, tobacco, and green bean leaves, respectively, caused by the indicated strains during a time course of infection using mycelial plugs. (**F**) Quantification of the lesion sizes caused by the indicated strains, as shown in (**A**). Left: spore inoculation; right: mycelial plug inoculation. **, ***: *p* < 0.01, 0.001. (**G**) Quantification of the lesion sizes caused by the indicated strains shown in (**B**). Left: spore inoculation; right: mycelial plug inoculation. *, **, ***: *p* < 0.5, 0.01, 0.001. (**H**) Quantification of the lesion sizes caused by the indicated strains as shown in (**C**–**E**), respectively. **, ***: *p* < 0.01, 0.001.

To assess the role of CBC in *B. cinerea* virulence, we conducted pathogenicity assays with WT, Δ*Bccbp20-4*, Δ*Bccbp20-6*, and Δ*Bccbp20*-C strains on multiple hosts using mycelial plugs and conidial suspensions. Firstly, conidial suspensions of the WT, Δ*Bccbp20-4*, Δ*Bccbp20-6*, and Δ*Bccbp20*-C strains were inoculated onto detached bean and tobacco leaves (Figure 6A,B). The lesion sizes were subsequently measured at various time points (Figure 6F,G). The results showed significantly smaller lesions induced by Δ*Bccbp20-4* and Δ*Bccbp20-6* compared to the control strains. Similar findings were observed when using mycelial plugs for inoculation (Figure 6A,B,F,G).

Secondly, fungal plugs of the WT, Δ*Bccbp80-3*, Δ*Bccbp80-33*, and Δ*Bccbp80*-C strains were inoculated onto detached bean, apricot, and tobacco leaves (Figure 6C,D,E). The lesion sizes were measured at 32 and 60 hpi. The results indicated that the lesions caused by the *BcCBP80* deletion mutant were significantly smaller than those caused by the control strains (Figure 6H). These findings demonstrate that the CBC is essential for the pathogenicity of *B. cinerea*.

### 3.8. BcCBP20 and BcCBP80 Are Important for Stress Responses

Emerging evidence establishes the CBC as an important regulator of abiotic stress responses in eukaryotes. Given that phytopathogens encounter multifactorial environmental challenges during host invasion, we propose CBC-mediated stress adaptation as an important factor for full virulence in *B. cinerea*.

**Figure 7:**
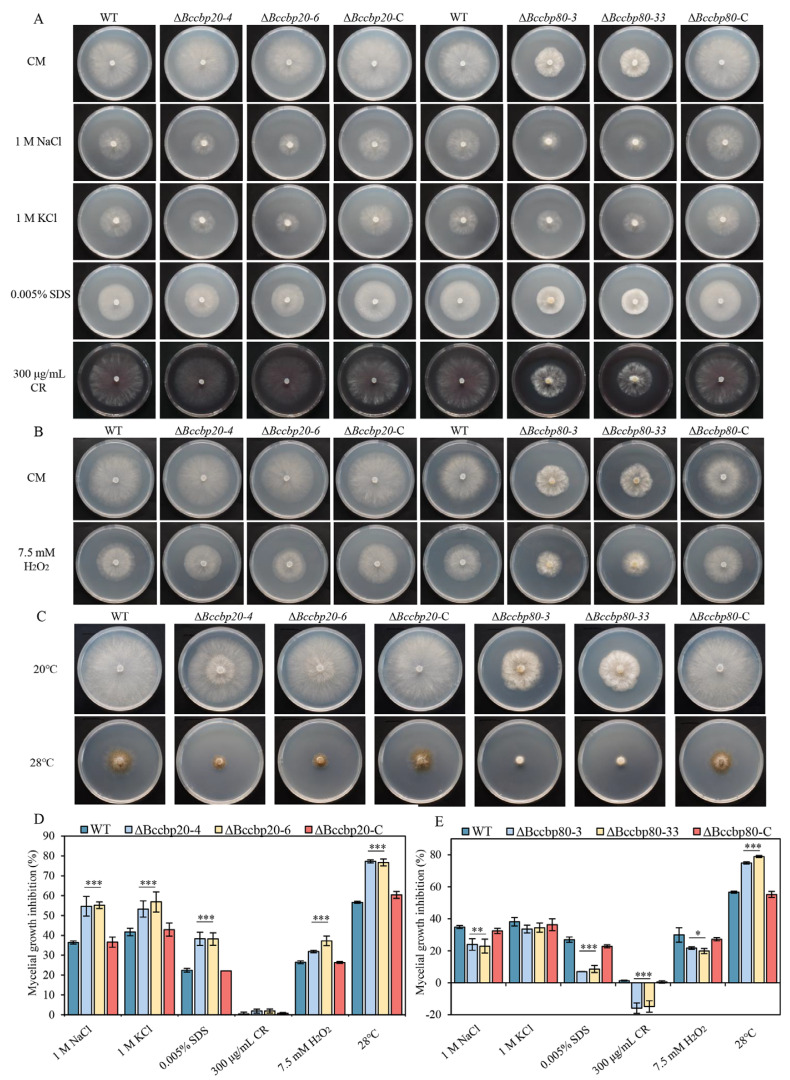
Growth and statistical analysis of the indicated strains under different stress conditions. (**A**) Development of mycelial plugs from the indicated strains on CM supplemented with the osmotic stress agents 1 M NaCl and 1 M KCl or cell wall-perturbing reagents sodium dodecyl sulfate (SDS, 0.005%) and Congo Red (CR, 300 µg/mL). (**B**) Development of mycelial plugs from the indicated strains on CM containing the oxidative stress agent hydrogen peroxide (H_2_O_2_, 7.5 mM). (**C**) Growth of mycelial plugs from the indicated strains on CM at 20 °C or 28 °C. (**D**,**E**) Statistical analysis of the relative mycelial growth inhibition observed in the indicated strains. *, **, ***: *p* < 0.05, 0.01, 0.001.

To dissect CBC’s role in stress tolerance, we subjected WT, Δ*Bccbp20*, Δ*Bccbp80*, and their complemented strains to combinatorial stress conditions on CM agar plates. The experimental stress conditions comprised salt stress (1 M NaCl/KCl), membrane and cell wall stress (0.005% SDS, 300 μg/mL Congo red (CR)), oxidative stress (7.5 mM H_2_O_2_), and temperature stress (20 °C vs. 28 °C) (Figure 7A,B,C). The results demonstrated that Δ*Bccbp20* mutants exhibited significantly enhanced sensitivity to these stressors. Conversely, the growth inhibition rates of Δ*Bccbp80* mutants were significantly lower than those of both WT and Δ*Bccbp80*-C strains on media containing 1 M NaCl, 0.005% SDS, 300 μg/mL CR, and 7.5 mM H_2_O_2_ but exhibited significantly higher growth inhibition at 28 °C compared to 20 °C (Figure 7D,E).

These findings underscore the crucial role of the CBC in modulating environmental stress responses, which may constitute the primary mechanism underlying the attenuated pathogenicity observed in Δ*Bccbp20* mutants.

### 3.9. BcCbp80 Is Essential for Infection Cushion Formation

The experimental data indicate that the reduced pathogenicity of Δ*Bccbp80* is not due to its impaired response to multiple environmental stressors. Instead, the formation of infection structures is crucial for *B. cinerea* pathogenicity. Consequently, we investigated the role of the CBC in this process.

**Figure 8:**
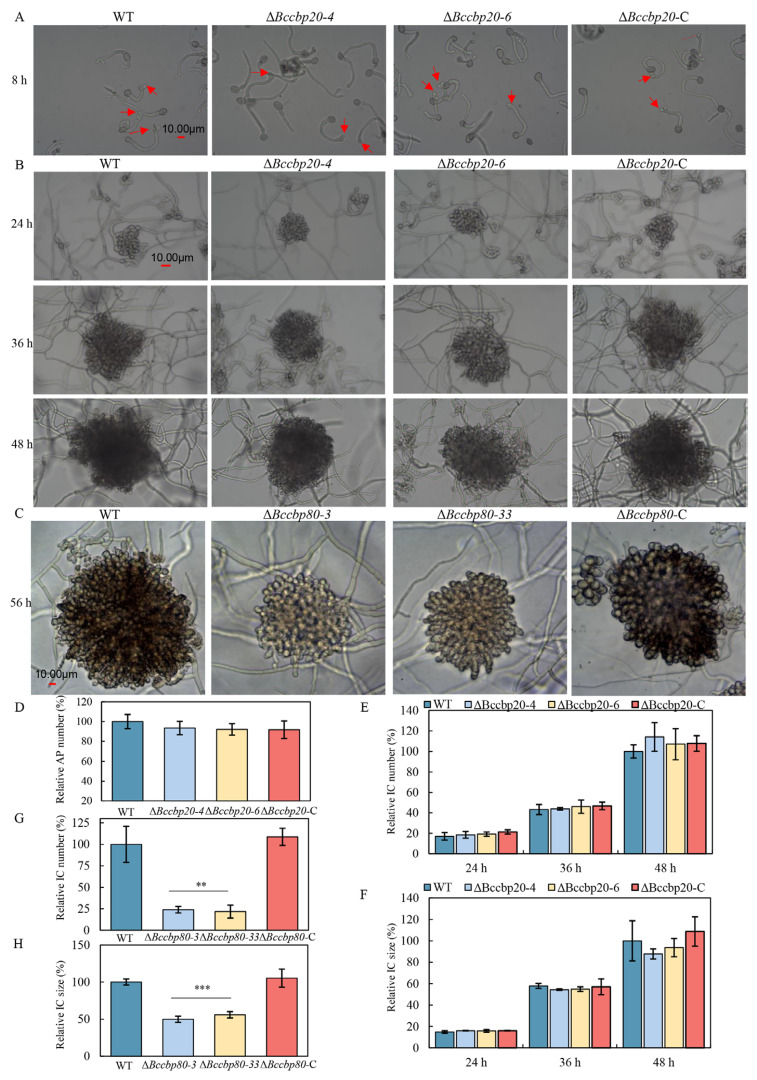
Development of infection structure in the indicated strains. (**A**) Appressoria formation in the indicated strains at 8 hpi. Red arrows indicate appressoria (AP). Scale bar = 10 µm. (**B**) Development of infection cushions in *B. cinerea* strains at the different time points post-inoculation as indicated. Scale bar = 10 µm. (**C**) Development of infection cushions in the indicated strains at 56 hpi. (**D**) Quantification of the relative number of appressoria in the indicated strains. (**E**,**G**) Quantification of the relative number of infection cushions in the indicated strains. (**F**–**H**) Quantification of the relative size of infection cushions in the indicated strains. **, ***: *p* < 0.01, 0.001.

Conidial suspensions of WT, Δ*Bccbp20-4*, Δ*Bccbp20-6*, and Δ*Bccbp20*-C strains were inoculated onto glass slides and incubated at 20 °C under high humidity in darkness. Appressoria formation occurred in all strains within 8 hpi, with no significant differences observed between strains (Figure 8A,D). For an infection cushion analysis, conidia were similarly inoculated and monitored at 24, 36, and 48 h intervals. Both size and quantity showed no significant variation between Δ*Bccbp20* mutants and controls (Figure 8B,E,F). Notably, fungal plugs of Δ*Bccbp80-3* and Δ*Bccbp80-33* mutants exhibited severe infection cushion defects at 56 hpi, showing a 50% size reduction and 75% quantity decrease compared to WT and complemented strains (Figure 8C,G,H).

These results establish *BcCBP80*’s predominant role over *BcCBP20* in infection structure formation, with structural deficiencies directly correlating with Δ*Bccbp80*’s reduced pathogenicity.

## 4. Discussion

In terms of interaction, the physical binding between Cbp20 and Cbp80 is highly conserved, from unicellular eukaryotes to mammals. However, there is very little experimental evidence to prove the interaction between them in filamentous pathogenic fungi. In *F. graminearum*, the physical interaction between FgCbp20 and FgCbp80 has only been experimentally validated using the yeast two-hybrid method. In *Neurospora crassa*, it has been demonstrated that NcCbp20 and NcCbp80 have a close interaction only through the utilization of Bimolecular Fluorescence Complementation [49]. Similarly, our study also confirmed this direct interaction between BcCbp20 and BcCbp80, using multiple methods in *B. cinerea*, including Y2H, GST-pull down, and SLC assays. Additionally, our experiments have further demonstrated that the interaction primarily relies on the N-terminus of BcCbp80 and the functional domain of BcCbp20. Our analysis has revealed that the MIF4G domain found in BcCbp80 is typically involved in forming protein complexes, which subsequently play pivotal roles in various stages of translation, including translation initiation and mRNA quality control processes. For example, the MIF4G domain is also present in eIF4G (eukaryotic initiation factor 4-gamma), a scaffold protein that is crucial in translation initiation by aiding in the recruitment of other translation factors to the 5’ cap structure of mRNA [50]. In contrast, BcCbp20 contains functional domains known as RNA Recognition Motifs (RRMs), which are critical regions for RNA-binding proteins to interact with RNA molecules. These RRMs can recognize and bind to specific sequences on RNA molecules, thereby playing a role in regulating the RNA lifecycle, including processes such as mRNA synthesis and maturation, the activation of non-coding RNA (ncRNA), splicing, and degradation [51]. In *B. cinerea*, the presence of functional domains is a crucial factor for the interactional conservation of BcCbp80 and BcCbp20 and also represents the key features for them to play a pivotal role in filamentous pathogenic fungi.

*CBP20* and *CBP80* play crucial roles, as evidenced by previous studies [23,24,25]. The evolutionarily conserved relationships across diverse species suggest that *BcCBP20* and *BcCBP80* also play important roles in *B. cinerea*. From our results, while CBC is vital for fungal growth and development, *BcCBP80* plays a more critical role than *BcCBP20* in hyphal growth and sporulation. This aligns with findings in *N. crassa*, where CBC has robust expression levels in vegetative and sexual cycles, and the loss of CBC impairs sexual development [49]. Intriguingly, studies in yeast reveal that CBC-deficient strains retain viability but suffer severe growth defects. This supports our observation of impaired fungal development in *B. cinerea* CBC mutants, which may similarly stem from disrupted splicing or the translation of ribosomal protein mRNAs. Such conserved phenotypes across fungi and yeast underscore CBC’s universal role in balancing RNA processing and translational fidelity—a critical nexus for cellular homeostasis [20,21].

CBC is capable of forming a microprocessor complex with the Dicer-like protein (DCL), which is responsible for processing pri-miRNA [14]. Wang et al. demonstrated that the host-induced gene silencing (HIGS) of *BcDCL1* and *BcDCL2* in *Arabidopsis* and tomato significantly silenced these fungal genes and suppressed *B. cinerea* virulence and hyphal growth. [52]. Bui et al. also demonstrated that the disruption of any CBCA complex subunit drastically attenuated the virulence of *F. graminearum* [48]. Our results indicate that both Δ*Bccbp20* and Δ*Bccbp80* significantly reduce pathogenicity towards the host as well. We show that BcCbp20 plays a crucial role in the response to environmental stress; this might be the key factor contributing to the reduced pathogenicity of BcCbp20. Much research has focused on the interplay between *CBP20/80* and stress responses in plants [26,27,28,29]. As we know, during host infection, fungi are subjected to a complex array of environmental stress factors, including temperature fluctuations, osmotic challenges, oxidative stress, and others [53].These stress factors interact with each other, collectively influencing the growth, development, and infection capabilities of fungi [54]. Hydrogen peroxide accumulation serves as a pivotal defense mechanism in plants against fungal invasions [55,56]. To counteract the oxidative stress induced by their hosts, pathogenic fungi engage in transcriptional, post-translational, and enzymatic responses to resist ROS toxicity and facilitate the adaptation to the host environment [37,57]. The Δ*Bccbp20* mutants exhibit sensitivity to H_2_O_2_ in vitro, which may hinder their ability to degrade ROS generated by the host during pathogen infection, subsequently diminishing their pathogenicity. Temperature is equally crucial for the growth and infection of fungi. *B. cinerea* exhibit varying degrees of adaptability to temperature, with extreme temperatures potentially inhibiting spore germination and mycelial growth, thereby affecting their pathogenicity [58]. The cell membrane, a critical cellular structure, is responsible for maintaining cell morphology and defending against external stressors [39,59]. Compared to the WT and complemented strains, the Δ*Bccbp20* mutants exhibit a higher sensitivity to the cell membrane-disrupting factor SDS, as well as to osmotic stress factors KCl and NaCl. This response to stress may also be the primary cause of vegetative growth defects and reduced germ tube length in Δ*Bccbp20* mutants. As we all know, *B. cinerea* primarily invades host plant cells or tissues via its infection structures, notably appressoria and infection cushions [60]. Regarding *BcCBP80*, although slower growth rates and abnormal mycelial morphology contribute to the reduced pathogenicity, the predominant factor lies in the decrease in both the quantity and size of infection cushions, which are critical for the establishment and progression of the infection process. There have been reports that the phenomenon of the reduction in the number and size of infection cushions leads to a decrease in the pathogenicity of mutants towards their hosts [36,61]. Pathogenicity assays conducted on ∆*Bccbp80* mutants using mycelial plugs demonstrate that the loss of *BcCBP80* in the pathogen significantly impairs the virulence of the ∆*Bccbp80* mutants, with the formation of infection cushions playing a pivotal role.

## 5. Conclusions

The utilization of continuously refined genetic transformation techniques to elucidate the functions of pathogenic genes in *B. cinerea* is crucial for identifying novel drug targets, thereby facilitating the development of innovative pesticides [62]. In this study, we found that BcCbp20 interacts with BcCbp80. The interaction specifically relies on the N-terminus of the BcCbp80 protein and the functional domain of BcCbp20. Functionally, the nucleus-localized *BcCBP20* and *BcCBP80* govern vegetative development and sporulation. Notably, Δ*Bccbp20* exhibits attenuated virulence likely associated with hypersensitivity to environmental stresses; whereas, Δ*Bccbp80* appears to affect pathogenicity through impaired infection cushion formation, among other factors. These results provide mechanistic insights into CBC-dependent regulatory circuits in *B. cinerea* and establish a foundation for developing CBC-targeted disease management strategies.

## Data Availability

The original contributions presented in this study are included in the article Supplementary Materials. Further inquiries can be directed to the corresponding author.

## Supplementary Materials

The following supporting information can be downloaded at: https://www.mdpi.com/article/10.3390/jof11060429/s1, Figure S1. Sequence and functional domain analysis of Cbp20 from various organisms. (A) Phylogenetic trees of Cbp20 (left) and its functional domain (right) from multiple organisms, including *Fusarium graminearum*, *Trichoderma reesei*, *Pyricularia oryzae*, *Neurospora crassa*, *Botrytis cinerea*, *Sclerotinia sclerotiorum*, *Aspergillus nidulans*, *Saccharomyces cerevisiae*, *Caenorhabditis elegans*, A*rabidopsis thaliana*, *Drosophila melanogaster*, and *Homo sapiens*, are listed in order. (B) Protein sequence alignment of Cbp20 in the species. The box indicates the RRM (RNA Recognition Motif) domain of BcCBP20; Figure S2. Sequence and functional domain analysis of Cbp80 from various organisms.(A) Phylogenetic trees of Cbp80 (left) and its functional domain (right) from multiple organisms, including *Fusarium graminearum*, *Trichoderma reesei*, *Pyricularia oryzae*, *Neurospora crassa*, *Botrytis cinerea*, *Sclerotinia sclerotiorum*, *Aspergillus nidulans*, *Saccharomyces cerevisiae*, *Caenorhabditis elegans*, *Arabidopsis thaliana*, *Drosophila melanogaster*, and *Homo sapiens*, are listed in order. (B) Protein sequence alignment of Cbp80 in the species. The red line represents MIF4G, the yellow line represents MIF4G_like, and the green line represents MIF4G_like_2 domains; Figure S3. BcCbp20 ^54-127^ physically interacts with N-terminal regions of BcCbp80. (A) Y2H assay mapping interaction domains. The N-terminal regions of BcCbp80 (aa 1-370 and 1-577) interact with full-length BcCbp20 and its functional domain (aa 54-127). (B) SLC assay validating interactions between BcCbp80 ^1-370^/BcCbp80 ^1-577^ and BcCbp20/BcCbp20 ^54-127^. (C) GST pull-down showing binding between BcCbp80 N-terminal fragments (aa 1-370: 41 kDa; aa 1-577: 65 kDa) and BcCbp20 domains (full-length: 47 kDa; aa 54-127: 32 kDa). GST control (26 kDa) is indicated. (D) Schematic representation of functional domains: BcCbp20 ^54-127^ (aa 54-127), BcCbp80 ^1-370^ (aa 1-370), BcCbp80 ^271-577^ (aa 271-577), BcCbp80 ^560-876^ (aa 560--876), BcCbp80 ^1-577^ (aa 1-577), and BcCbp80 ^271-876^ (aa 271-876); Figure S4. Schematic illustration of gene knockout, complementation, and identification of transformants. (A) Strategy for generation of BcCBP20 gene disruption (∆Bccbp20) mutant strains. (B) Strategy for generation of BcCBP20 gene complemented (∆Bccbp20-C) strains. (C) Strategy for generation of BcCBP80 gene disruption (∆Bccbp80) mutant strains. (D) Strategy for generation of BcCBP80 gene complemented (∆Bccbp80-C) strains. (E) Schematic diagram of PCR primers used for the identification of ∆Bccbp20 mutant strains. (F) Identification of ∆Bccbp20 and ∆Bccbp20-C transformants. The upper panel displays the results of a diagnostic PCR utilizing four primer pairs: 1 (20-nb-F/20-nb-R), 2 (HY-F/YG-R), 3 (20-PF/YG-R), and 4 (HY-F/20-PR). This PCR yields distinguishable product sizes: 706 bp for the wild-type (WT) using 20-nb-F/20-nb-R, and 476 bp (HY-F/YG-R), 1984 bp (20-PF/YG-R), and 1556 bp (HY-F/20-PR) for the transformants. The lower panel reveals the detection of BcCBP20 expression in the WT, ∆Bccbp20 mutants, and ∆Bccbp20-C complemented strain, as determined via a RT-PCR analysis. (G) Schematic diagram of PCR primers for the identification of ∆Bccbp80 mutant strains. (H) Identification of ∆Bccbp80 and ∆Bccbp80-C transformants. The upper panel displays the results of a diagnostic PCR utilizing four primer pairs: 1 (80-nb-F/80-nb-R), 2 (HY-F/YG-R), 3 (80-PF/YG-R), and 4 (HY-F/80-PR). This PCR yields distinguishable product sizes: 442 bp for the WT using 80-nb-F/80-nb-R, and 476 bp (HY-F/YG-R), 2282 bp (80-PF/YG-R), and 1802 bp (HY-F/80-PR) for the transformants. The lower panel reveals the detection of BcCBP80 expression in the WT, ∆Bccbp80 mutants, and ∆Bccbp80-C complemented strain, as determined by qRT-PCR; Table S1: Primers and sequence used in this study; Table S2: proteins-Test--Control--.

## References

[1] Fillinger S., Elad Y. (2016). Botrytis—The Fungus, the Pathogen and its Management in Agricultural Systems.

[2] De Miccolis Angelini R.M., Rotolo C., Masiello M., Gerin D., Pollastro S., Faretra F. (2014). Occurrence of fungicide resistance in populations of *Botryotinia fuckeliana* (*Botrytis cinerea*) on table grape and strawberry in southern Italy. Pest Manag. Sci..

[3] Sun H.Y., Wang H.C., Chen Y., Li H.X., Zhou M.G. (2010). Multiple Resistance of *Botrytis cinerea* from Vegetable Crops to Carbendazim, Diethofencarb, Procymidone, and Pyrimethanil in China. Plant Dis..

[4] Richardson P.M., Amselem J., Cuomo C.A., van Kan J.A.L., Viaud M., Benito E.P., Couloux A., Coutinho P.M., de Vries R.P., Dyer P.S. (2011). Genomic Analysis of the Necrotrophic Fungal Pathogens *Sclerotinia sclerotiorum* and *Botrytis cinerea*. PLoS Genet..

[5] Williamson B., Tudzynski B., Tudzynski P., van Kan J.A. (2007). *Botrytis cinerea*: The cause of grey mould disease. Mol. Plant Pathol..

[6] Gourgues M., Brunet-Simon A., Lebrun M.H., Levis C. (2004). The tetraspanin BcPls1 is required for appressorium-mediated penetration of *Botrytis cinerea* into host plant leaves. Mol. Microbiol..

[7] Choquer M., Rascle C., Gonçalves I.R., de Vallée A., Ribot C., Loisel E., Smilevski P., Ferria J., Savadogo M., Souibgui E. (2021). The infection cushion of *Botrytis cinerea*: A fungal ‘weapon’ of plant-biomass destruction. Environ. Microbiol..

[8] Weiberg A., Wang M., Lin F.M., Zhao H., Zhang Z., Kaloshian I., Huang H.D., Jin H. (2013). Fungal small RNAs suppress plant immunity by hijacking host RNA interference pathways. Science.

[9] Colmenares A.J., Aleu J., Durán-Patrón R., Collado I.G., Hernández-Galán R. (2002). The Putative Role of Botrydial and Related Metabolites in the Infection Mechanism of *Botrytis cinerea*. J. Chem. Ecol..

[10] González-Fernández R., Valero-Galván J., Gómez-Gálvez F.J., Jorrín-Novo J.V. (2015). Unraveling the in vitro secretome of the phytopathogen *Botrytis cinerea* to understand the interaction with its hosts. Front. Plant Sci..

[11] Kataoka N. (2024). The Nuclear Cap-Binding Complex, a multitasking binding partner of RNA polymerase II transcripts. J. Biochem..

[12] Görnemann J., Kotovic K.M., Hujer K., Neugebauer K.M. (2005). Cotranscriptional spliceosome assembly occurs in a stepwise fashion and requires the cap binding complex. Mol. Cell.

[13] Gruber J.J., Zatechka D.S., Sabin L.R., Yong J., Lum J.J., Kong M., Zong W.X., Zhang Z., Lau C.K., Rawlings J. (2009). Ars2 links the nuclear cap-binding complex to RNA interference and cell proliferation. Cell.

[14] Kim S., Yang J.Y., Xu J., Jang I.C., Prigge M.J., Chua N.H. (2008). Two Cap-Binding Proteins CBP20 and CBP80 are Involved in Processing Primary MicroRNAs. Plant Cell Physiol..

[15] Ryu I., Kim Y.K. (2017). Translation initiation mediated by nuclear cap-binding protein complex. BMB Rep..

[16] Grudzien E., Kalek M., Jemielity J., Darzynkiewicz E., Rhoads R.E. (2006). Differential Inhibition of mRNA Degradation Pathways by Novel Cap Analogs. J. Biol. Chem..

[17] Nojima T., Hirose T., Kimura H., Hagiwara M. (2007). The interaction between cap-binding complex and RNA export factor is required for intronless mRNA export. J. Biol. Chem..

[18] Gebhardt A., Habjan M., Benda C., Meiler A., Haas D.A., Hein M.Y., Mann A., Mann M., Habermann B., Pichlmair A. (2015). mRNA export through an additional cap-binding complex consisting of NCBP1 and NCBP3. Nat. Commun..

[19] Gonatopoulos-Pournatzis T., Cowling V.H. (2013). Cap-binding complex (CBC). Biochem. J..

[20] Fortes P., Kufel J., Fornerod M., Polycarpou-Schwarz M., Lafontaine D., Tollervey D., Mattaj I.W. (1999). Genetic and Physical Interactions Involving the Yeast Nuclear Cap-Binding Complex. Mol Cell Biol..

[21] Das B., Guo Z., Russo P., Chartrand P., Sherman F. (2000). The Role of Nuclear Cap Binding Protein Cbc1p of Yeast in mRNA Termination and Degradation. Mol. Cell. Biol..

[22] Hossain M.A., Claggett J.M., Nguyen T., Johnson T.L. (2009). The cap binding complex influences H2B ubiquitination by facilitating splicing of the *SUS1* pre-mRNA. RNA.

[23] Katahira J., Ohmae T., Yasugi M., Sasaki R., Itoh Y., Kohda T., Hieda M., Yokota Hirai M., Okamoto T., Miyamoto Y. (2023). Nsp14 of SARS-CoV-2 inhibits mRNA processing and nuclear export by targeting the nuclear cap-binding complex. Nucleic Acids Res..

[24] Singh G., Seufzer B., Song Z., Zucko D., Heng X., Boris-Lawrie K. (2021). HIV-1 hypermethylated guanosine cap licenses specialized translation unaffected by mTOR. Proc. Nat. Acad. Sci. USA.

[25] Bier K., York A., Fodor E. (2011). Cellular cap-binding proteins associate with influenza virus mRNAs. J. Gen. Virol..

[26] Hugouvieux V., Kwak J.M., Schroeder J.I. (2001). An mRNA cap binding protein, ABH1, modulates early abscisic acid signal transduction in *Arabidopsis*. Cell.

[27] Kong X.X., Ma L., Yang L.M., Chen Q., Xiang N., Yang Y.P., Hu X.Y. (2014). Quantitative Proteomics Analysis Reveals That the Nuclear Cap-Binding Complex Proteins CBP20 and CBP80 Modulate the Salt Stress Response. J. Proteome Res..

[28] Li Y., Guo Q.H., Liu P., Huang J.G., Zhang S.Z., Yang G.D., Wu C.G., Zheng C.C., Yan K. (2021). Dual roles of the serine/arginine-rich splicing factor SR45a in promoting and interacting with nuclear cap-binding complex to modulate the salt-stress response in *Arabidopsis*. New Phytol..

[29] Zhang F., Wang L.K., Lim J.Y., Kim T., Pyo Y., Sung S., Shin C., Qiao H. (2016). Phosphorylation of CBP20 Links MicroRNA to Root Growth in the Ethylene Response. PLoS Genet..

[30] Tamura K., Peterson D., Peterson N., Stecher G., Nei M., Kumar S. (2011). MEGA5: Molecular Evolutionary Genetics Analysis Using Maximum Likelihood, Evolutionary Distance, and Maximum Parsimony Methods. Mol. Biol. Evol..

[31] Feng H.Q., Li G.H., Du S.W., Yang S., Li X.Q., de Figueiredo P., Qin Q.M. (2017). The septin protein Sep4 facilitates host infection by plant fungal pathogens via mediating initiation of infection structure formation. Environ. Microbiol..

[32] Zhang Y., Jia C., Liu Y., Li G., Li B., Shi W., Zhang Y., Hou J., Qin Q., Zhang M. (2024). The Fungal Transcription Factor BcTbs1 from *Botrytis cinerea* Promotes Pathogenicity via Host Cellulose Degradation. J. Agric. Food Chem..

[33] Zeng L.M., Zhang J., Han Y.C., Yang L., Wu M.D., Jiang D.H., Chen W.D., Li G.Q. (2016). Degradation of oxalic acid by the mycoparasite Coniothyrium minitans plays an important role in interacting with *Sclerotinia sclerotiorum*. Environ. Microbiol..

[34] Tang J.J., Wu M.D., Zhang J., Li G.Q., Yang L. (2021). *Botrytis cinerea* G Protein β Subunit Bcgb1 Controls Growth, Development and Virulence by Regulating cAMP Signaling and MAPK Signaling. J. Fungi.

[35] Sun J., Sun C.H., Chang H.W., Yang S., Liu Y., Zhang M.Z., Hou J., Zhang H., Li G.H., Qin Q.M. (2021). Cyclophilin BcCyp2 Regulates Infection-Related Development to Facilitate Virulence of the Gray Mold Fungus *Botrytis cinerea*. Int. J. Mol. Sci..

[36] Liu Y., Liu J.K., Li G.H., Zhang M.Z., Zhang Y.Y., Wang Y.Y., Hou J., Yang S., Sun J., Qin Q.M. (2019). A novel *Botrytis cinerea*-specific gene *BcHBF1* enhances virulence of the grey mould fungus via promoting host penetration and invasive hyphal development. Mol. Plant Pathol..

[37] Cao S.N., Yuan Y., Qin Y.H., Zhang M.Z., de Figueiredo P., Li G.H., Qin Q.M. (2018). The pre-rRNA processing factor Nop53 regulates fungal development and pathogenesis via mediating production of reactive oxygen species. Environ. Microbiol..

[38] Rolland S., Jobic C., Fèvre M., Bruel C. (2003). *Agrobacterium*-mediated transformation of *Botrytis cinerea*, simple purification of monokaryotic transformants and rapid conidia-based identification of the transfer-DNA host genomic DNA flanking sequences. Curr. Genet..

[39] Yang S., Sun J., Xue A., Li G., Sun C., Hou J., Qin Q.-M., Zhang M. (2024). Novel *Botrytis cinerea* Zn(II)2Cys6 Transcription Factor BcFtg1 Enhances the Virulence of the Gray Mold Fungus by Promoting Organic Acid Secretion and Carbon Source Utilization. J. Agric. Food Chem..

[40] Giesbert S., Schumacher J., Kupas V., Espino J., Segmüller N., Haeuser-Hahn I., Schreier P.H., Tudzynski P. (2012). Identification of pathogenesis-associated genes by T-DNA-mediated insertional mutagenesis in *Botrytis cinerea*: A type 2A phosphoprotein phosphatase and an *SPT3* transcription factor have significant impact on virulence. Mol. Plant. Microbe Interact..

[41] Hou J., Feng H.Q., Chang H.W., Liu Y., Li G.H., Yang S., Sun C.H., Zhang M.Z., Yuan Y., Sun J. (2020). The H3K4 demethylase Jar1 orchestrates ROS production and expression of pathogenesis-related genes to facilitate *Botrytis cinerea* virulence. New Phytol..

[42] Zhang Y.S., Wang L.M., Liang S., Zhang P.P., Kang R.J., Zhang M.J., Wang M., Chen L.L., Yuan H.X., Ding S.L. (2020). FpDep1, a component of Rpd3L histone deacetylase complex, is important for vegetative development, ROS accumulation, and pathogenesis in *Fusarium pseudograminearum*. Fungal Genet. Biol..

[43] Shi X.T., Xiong Y.H., Zhang K., Zhang Y.S., Zhang J.Q., Zhang L.L., Xiao Y.T., Wang G.L., Liu W.D. (2023). The ANIP1-OsWRKY62 module regulates both basal defense and Pi9-mediated immunity against *Magnaporthe oryzae* in rice. Mol. Plant.

[44] Shi X.T., Xie X., Guo Y.W., Zhang J.Q., Gong Z.W., Zhang K., Mei J., Xia X.Y., Xia H.X., Ning N. (2024). A fungal core effector exploits the OsPUX8B.2–OsCDC48-6 module to suppress plant immunity. Nat. Commun..

[45] Li Z.Q., Wu L.Y., Wu H., Zhang X.X., Mei J., Zhou X.P., Wang G.L., Liu W.D. (2020). Arginine methylation is required for remodelling pre-mRNA splicing and induction of autophagy in rice blast fungus. New Phytol..

[46] Shi X.T., Long Y., He F., Zhang C.Y., Wang R.Y., Zhang T., Wu W., Hao Z.Y., Wang Y., Wang G.L. (2018). The fungal pathogen suppresses innate immunity by modulating a host potassium channel. PLoS Pathog..

[47] Ding H., Zhou Y., Wang H. (2019). Development of an indirect ELISA for detecting humoral immunodominant proteins of *Mycoplasma hyopneumoniae* which can discriminate between inactivated bacterin-induced hyperimmune sera and convalescent sera. BMC Vet. Res..

[48] Bui D.C., Kim J.E., Shin J., Lim J.Y., Choi G.J., Lee Y.W., Seo J.A., Son H. (2019). *ARS2* Plays Diverse Roles in DNA Damage Response, Fungal Development, and Pathogenesis in the Plant Pathogenic Fungus *Fusarium graminearum*. Front. Microbiol..

[49] Decker L.M., Xiao H., Boone E.C., Vierling M.M., Shanker B.S., Kingston S.L., Boone S.F., Haynes J.B., Shiu P.K.T. (2017). The Nuclear Cap-Binding Complex Mediates Meiotic Silencing by Unpaired DNA. G3.

[50] Ponting C.P. (2000). Novel eIF4G domain homologues linking mRNA translation with nonsense-mediated mRNA decay. Trends Biochem. Sci..

[51] Maris C., Dominguez C., Allain F.H.T. (2005). The RNA recognition motif, a plastic RNA-binding platform to regulate post-transcriptional gene expression. FEBS J..

[52] Wang M., Weiberg A., Lin F.M., Thomma B.P.H.J., Huang H.D., Jin H. (2016). Bidirectional cross-kingdom RNAi and fungal uptake of external RNAs confer plant protection. Nat. Plants.

[53] Segmüller N., Ellendorf U., Tudzynski B., Tudzynski P. (2007). *BcSAK1*, a Stress-Activated Mitogen-Activated Protein Kinase, Is Involved in Vegetative Differentiation and Pathogenicity in *Botrytis cinerea*. Eukaryot. Cell.

[54] Luis C., Verónica P., Luis F.L., Paulo C. (2017). Recent Advances in the Study of the Plant Pathogenic Fungus *Botrytis cinerea* and its Interaction with the Environment. Curr. Protein Pept. Sci..

[55] Mellersh D.G., Foulds I.V., Higgins V.J., Heath M.C. (2002). H_2_O_2_ plays different roles in determining penetration failure in three diverse plant-fungal interactions. Plant J..

[56] Shetty N.P., Mehrabi R., Lütken H., Haldrup A., Kema G.H.J., Collinge D.B., Jørgensen H.J.L. (2007). Role of hydrogen peroxide during the interaction between the hemibiotrophic fungal pathogen *Septoria tritici* and wheat. New Phytol..

[57] Warris A., Ballou E.R. (2019). Oxidative responses and fungal infection biology. Semin. Cell Dev. Biol..

[58] Li T., Liu R., Liu Z., Chang J., Li J. (2023). Effects of Intermittent Temperature and Humidity Regulation on Tomato Gray Mold. Plant Dis..

[59] Morgan M.J., Kim Y.S., Liu Z. (2007). Lipid Rafts and Oxidative Stress–Induced Cell Death. Antioxid. Redox Signal..

[60] Choquer M., Fournier E., Kunz C., Levis C., Pradier J.M., Simon A., Viaud M. (2007). *Botrytis cinerea* virulence factors: New insights into a necrotrophic and polyphageous pathogen. FEMS Microbiol. Lett..

[61] Liu N., Ren W.C., Li F.J., Chen C.J., Ma Z.H. (2019). Involvement of the cysteine protease BcAtg4 in development and virulence of *Botrytis cinerea*. Curr. Genet..

[62] Tang M.Y., Wang Y.Y.Z., Wang K.X., Zhou Y.H., Zhao E.S., Zhang H., Zhang M.Z., Yu H., Zhao X., Li G. (2024). Codon Optimization Enables the Geneticin Resistance Gene to Be Applied Efficiently to the Genetic Manipulation of the Plant Pathogenic Fungus *Botrytis cinerea*. Plants.

