# Supplementary material for: CBC Complex Regulates Hyphal Growth, Sclerotial Quantity, and Pathogenicity in the Necrotrophic Fungus Botrytis cinerea
Source: J Fungi (Basel). 2025 Jun 2;11(6):429. doi: 10.3390/jof11060429 (PMC12194085; doi:10.3390/jof11060429)
Supplement: Supplementary file 1 [file jof-11-00429-s001.zip › Supporting information.pdf]

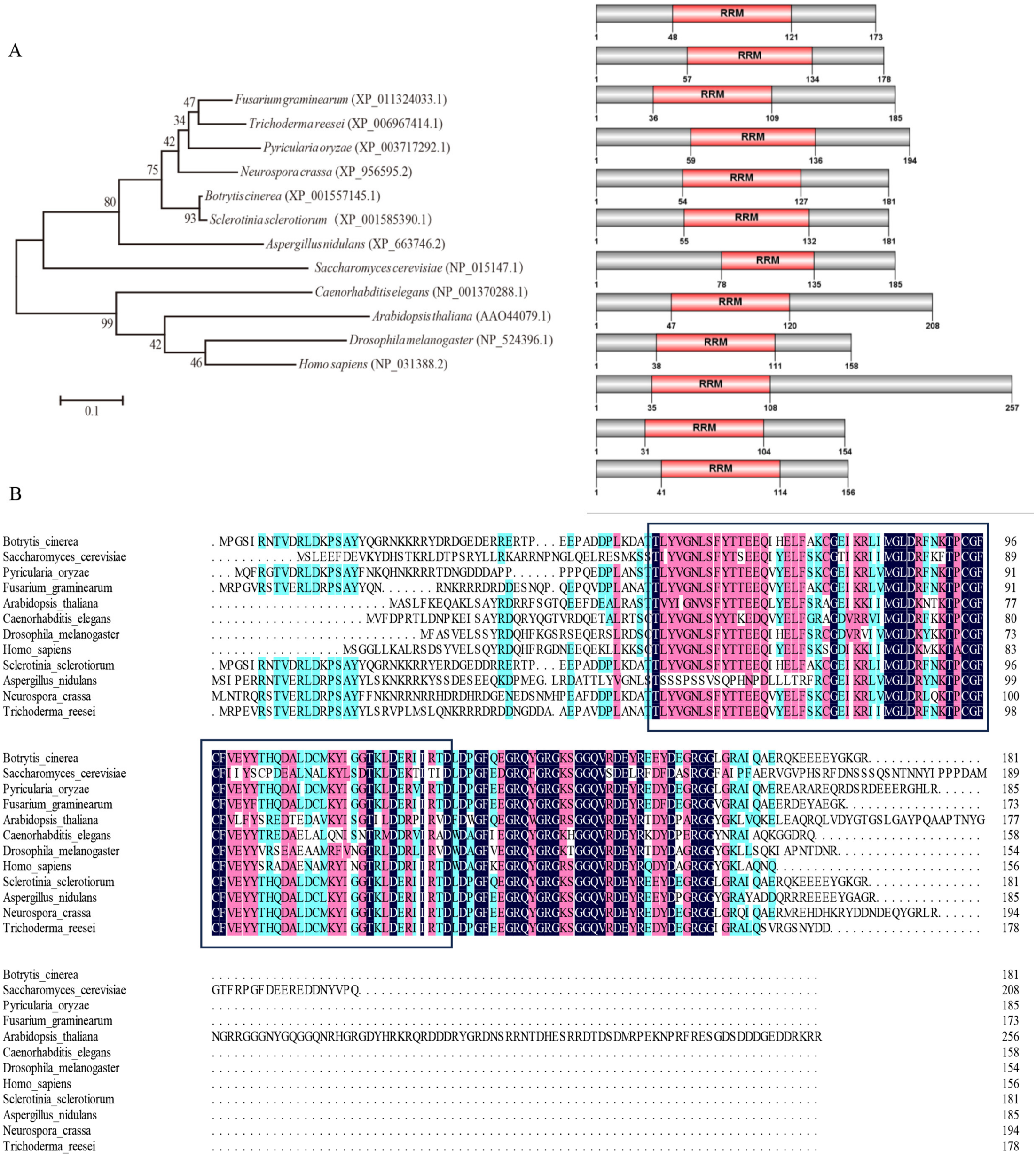

Figure S1. Sequence and functional domain analysis of Cbp20 from various organisms. (A) Phylogenetic trees of Cbp20 (left) and its functional domain (right) from multiple organisms, including *Fusarium graminearum, Trichoderma reesei, Pyricularia oryzae, Neurospora crassa, Botrytis cinerea*, *Sclerotinia sclerotiorum, Aspergillus nidulans, Saccharomyces cerevisiae*, *Caenorhabditis elegans*, *Arabidopsis thaliana*, *Drosophila melanogaster*, and *Homo sapiens*, are listed in order. (B) Protein sequence alignment of Cbp20 in the species. The box indicates the RRM (RNA Recognition Motif) domain of *BcCBP20*.

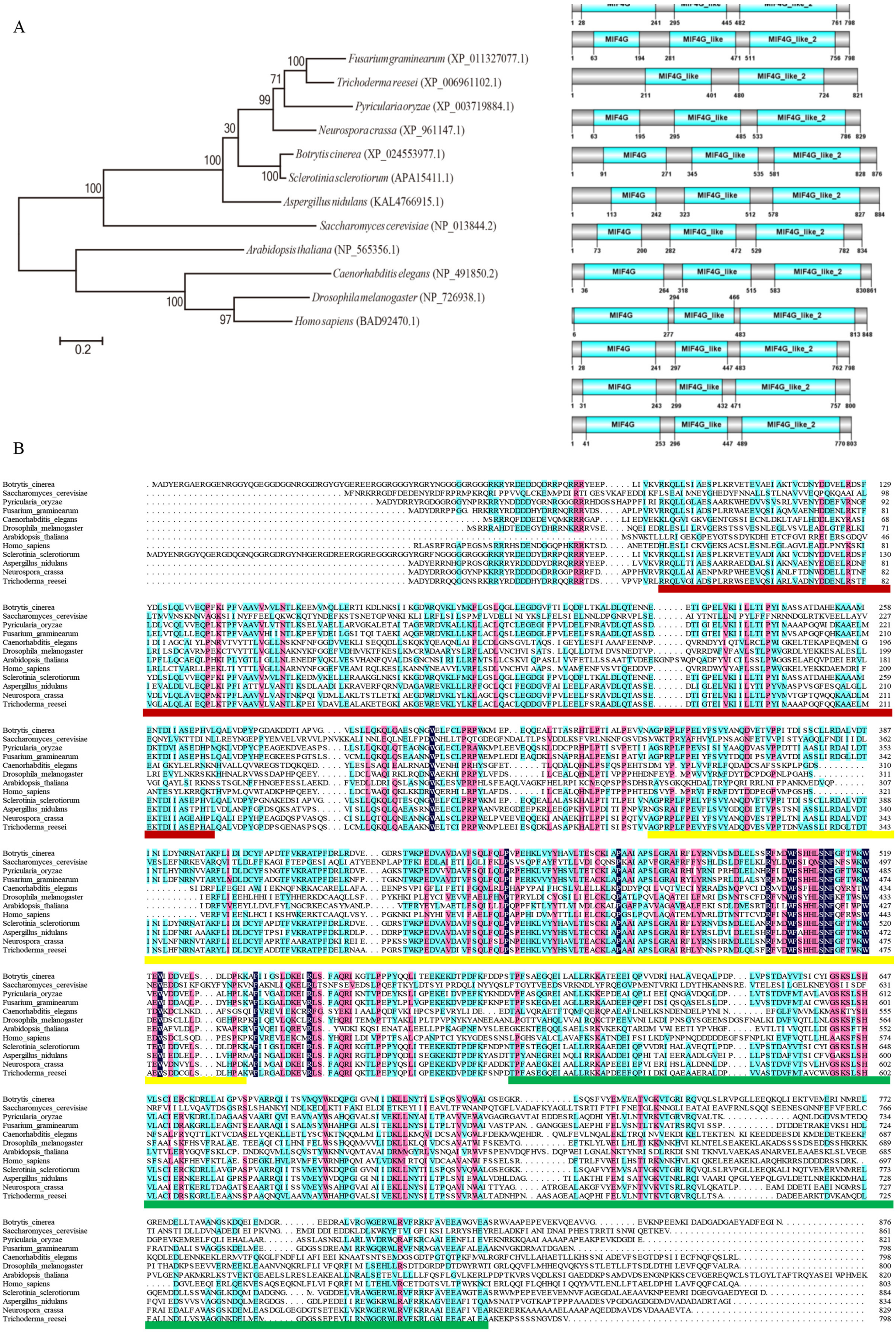

Figure S2. Sequence and functional domain analysis of Cbp80 from various organisms. (A) Phylogenetic trees of Cbp80 (left) and its functional domain (right) from multiple organisms, including *Fusarium graminearum, Trichoderma reesei, Pyricularia oryzae,*

Neurospora crassa, Botrytis cinerea, *Sclerotinia sclerotiorum, Aspergillus nidulans, Saccharomyces cerevisiae*, *Caenorhabditis elegans*, *Arabidopsis thaliana*, *Drosophila melanogaster*, and *Homo sapiens*, are listed in order. (B) Protein sequence alignment of Cbp80 in the species. The red line represents MIF4G, the yellow line represents MIF4G_like, and the green line represents MIF4G_like_2 domains.

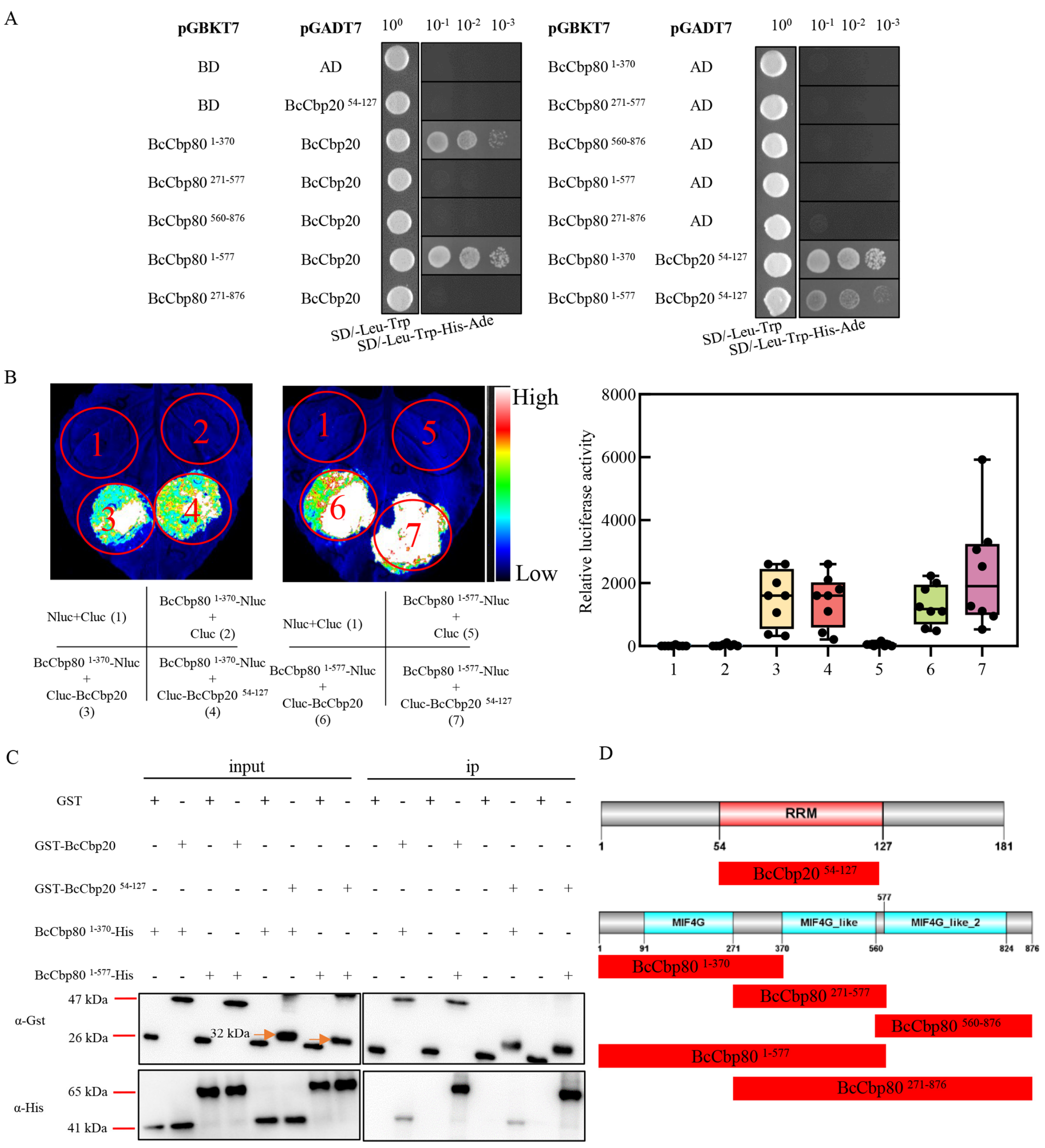

Figure S3. BcCbp20 $^{54\text{-}127}$ physically interacts with N-terminal regions of BcCbp80. (A) Y2H assay mapping interaction domains. The N-terminal regions of BcCbp80 (aa 1-370 and 1-577) interact with full-length BcCbp20 and its functional domain (aa 54-127). (B) SLC assay

validating interactions between BcCbp80 $^{1-370}$/BcCbp80 $^{1-577}$ and BcCbp20/BcCbp20 $^{54-127}$. (C) GST pull-down showing binding between BcCbp80 N-terminal fragments (aa 1-370: 41 kDa; aa 1-577: 65 kDa) and BcCbp20 domains (full-length: 47 kDa; aa 54-127: 32 kDa). GST control (26 kDa) is indicated. (D) Schematic representation of functional domains: BcCbp20 $^{54-127}$ (aa 54-127), BcCbp80 $^{1-370}$ (aa 1-370), BcCbp80 $^{271-577}$ (aa 271-577), BcCbp80 $^{560-876}$ (aa 560--876), BcCbp80 $^{1-577}$ (aa 1-577), and BcCbp80 $^{271-876}$ (aa 271-876).

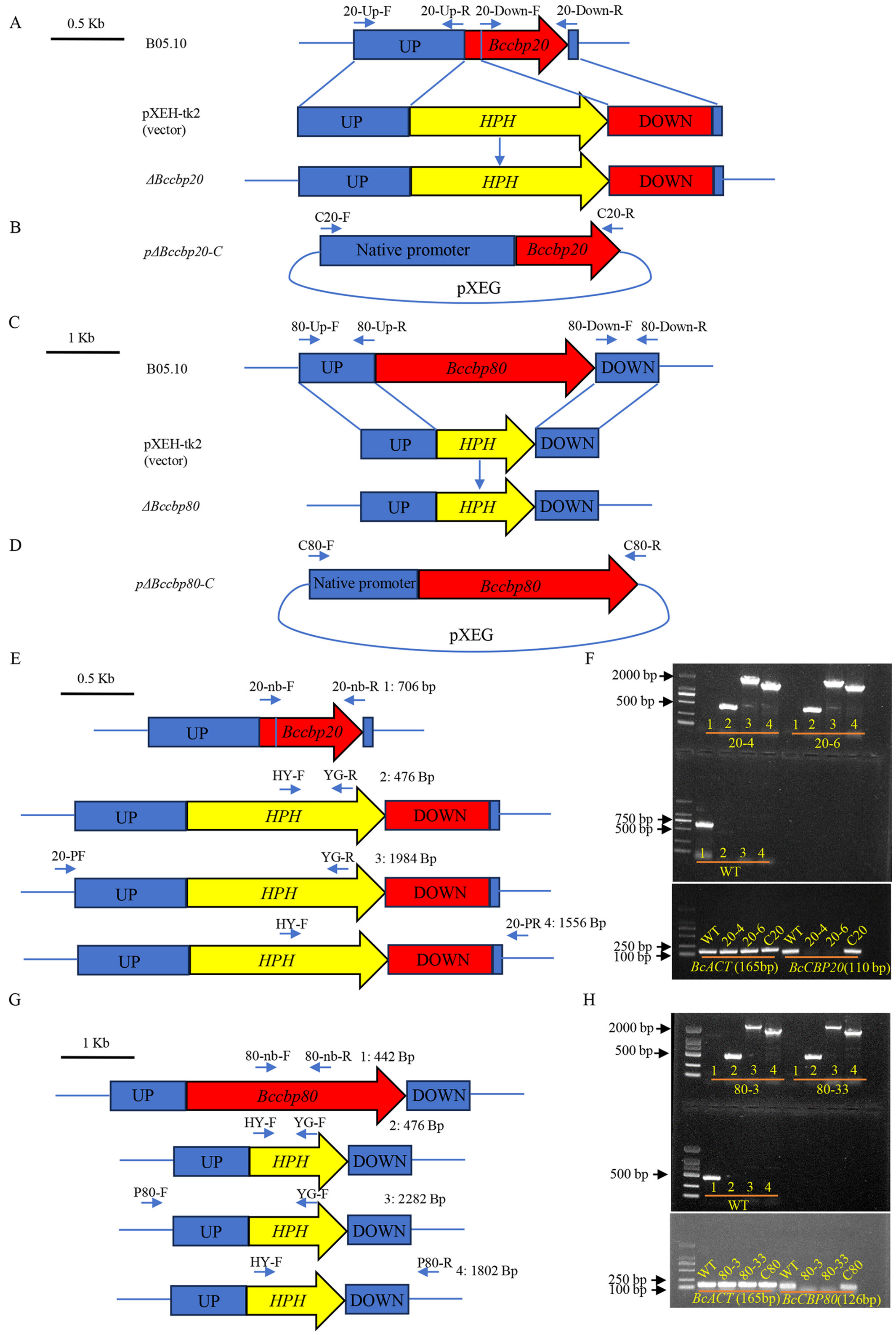

Figure S4. Schematic illustration of gene knockout, complementation, and identification of transformants.

(A) Strategy for generation of *BcCBP20* gene disruption (Δ*Bccbp20*) mutant strains.

(B) Strategy for generation of *BcCBP20* gene complemented (ΔB*ccbp20*-C) strains.

(C) Strategy for generation of *BcCBP80* gene disruption (Δ*Bccbp80*) mutant strains.

(D) Strategy for generation of *BcCBP80* gene complemented (ΔB*ccbp80*-C) strains.

(E) Schematic diagram of PCR primers used for the identification of Δ*Bccbp20* mutant strains.

(F) Identification of Δ*Bccbp20* and Δ*Bccbp20*-C transformants. The upper panel displays the results of a diagnostic PCR utilizing four primer pairs: 1 (20-nb-F/20-nb-R), 2 (HY-F/YG-R), 3 (20-PF/YG-R), and 4 (HY-F/20-PR). This PCR yields distinguishable product sizes: 706 bp for the wild-type (WT) using 20-nb-F/20-nb-R, and 476 bp (HY-F/YG-R), 1984 bp (20-PF/YG-R), and 1556 bp (HY-F/20-PR) for the transformants. The lower panel reveals the detection of *BcCBP20* expression in the WT, Δ*Bccbp20* mutants, and Δ*Bccbp20*-C complemented strain, as determined via a RT-PCR analysis.

(G) Schematic diagram of PCR primers for the identification of Δ*Bccbp80* mutant strains.

(H) Identification of Δ*Bccbp80* and Δ*Bccbp80*-C transformants. The upper panel displays the results of a diagnostic PCR utilizing four primer pairs: 1 (80-nb-F/80-nb-R), 2 (HY-F/YG-R), 3 (80-PF/YG-R), and 4 (HY-F/80-PR). This PCR yields distinguishable product sizes: 442 bp for the WT using 80-nb-F/80-nb-R, and 476 bp (HY-F/YG-R), 2282 bp (80-PF/YG-R), and 1802 bp (HY-F/80-PR) for the transformants. The lower panel reveals the detection of *BcCBP80 expression* in the WT, Δ*Bccbp80* mutants, and Δ*Bccbp80*-C complemented strain, as determined by qRT-PCR.
